# Remote evaluation of rice nitrogen utilization efficiency using chlorophyll-related spectral indices derived from unmanned aerial vehicle imagery

**DOI:** 10.3389/fpls.2026.1851420

**Published:** 2026-05-28

**Authors:** Xiaojuan Liu, Shenghui Fang, Fangting Li, Si Liang, Yuyan Yan, Qiang Xiong, Xianting Wu, Yi Peng

**Affiliations:** 1 School of Remote Sensing and Information Engineering, Wuhan University, Wuhan, China; 2 State Key Laboratory of Information Engineering in Surveying, Mapping and Remote Sensing, Wuhan University, Wuhan, China; 3 Hubei Institute of Photogrammetry and Remote Sensing, Wuhan, China; 4 State Key Laboratory of Hybrid Rice, College of Life Sciences, Wuhan University, Wuhan, China

**Keywords:** chlorophyll-related indices, nitrogen contents, nitrogen utilization efficiency, rice, unmanned aerial vehicle image

## Abstract

**Introduction:**

Nitrogen utilization efficiency (NUtE) directly reflects the efficiency of nitrogen remobilization to grains, serving as a key indicator of yield formation and environmental performance. However, conventional methods for assessing NUtE rely on destructive sampling and laboratory analysis, which are labor-intensive and time-consuming, whereas most existing remote sensing studies estimate NUtE by directly regressing spectral features against the final efficiency value without decomposing it into its underlying physiological components.

**Methods:**

This study developed a remote-sensing-based indicator of rice NUtE based on chlorophyll-related vegetation indices at key rice growth stages, termed the Nitrogen Utilization Efficiency-Vegetation Index (NUtE-VI). NUtE showed a close and near-linear relationship with the ratio of panicle nitrogen accumulation from heading to dough stage (ΔPNA_dough-heading_, sink indicator) to leaf nitrogen accumulation at booting stage (LNA_booting_, source indicator). Therefore, with multi-site field experiments across different rice cultivars and nitrogen treatments, this study employed unmanned aerial vehicle imaging to accurately estimate rice leaf and panicle nitrogen accumulations, enabling rapid, large-scale evaluation of rice NUtE.

**Results:**

This proposed index showed a strong correlation with measured NUtE (R^2^ = 0.72, rRMSE = 10.84%) and effectively captured the distinct patterns of NUtE across different nitrogen treatments and cultivars.

**Discussion:**

Our developed indicator is generalizable across diverse conditions for high-throughput selection of nitrogen-efficient cultivars and precision nitrogen management in sustainable agriculture.

## 1 Introduction

Nitrogen is an essential macronutrient for plant growth and development, playing a key role in photosynthesis, energy metabolism, and biomass synthesis. To meet the increasing global demand for food production, nitrogen fertilization has been adopted as a routine and effective agricultural practice, which increases the availability of nitrogen in the soil, thereby enabling crops to take up more nitrogen, thus promoting growth and increasing yield (Huang et al., 2022). For rice, nitrogen is particularly critical because it directly affects tillering, panicle formation, and grain filling. However, excessive nitrogen application not only leads to environmental problems such as water eutrophication and greenhouse gas emissions but also inhibits crop growth due to nitrogen surplus, resulting in lodging, delayed maturity, and reduced yield (Greaver et al., 2016; Tufail et al., 2024; Zhang et al., 2017). Therefore, studies have been conducted to select or develop suitable rice cultivars that can absorb more nitrogen under reduced nitrogen input, thereby ensuring adequate nitrogen supply while protecting the environment (Chen, 2026; Yang et al., 2025).

Nitrogen use efficiency (NUE) is a widely used indicator for evaluating crop nitrogen utilization, yet it encompasses multiple calculation methods based on different reference bases. Some definitions focus on soil-based efficiency, relating grain yield or biomass to total nitrogen input (including both soil and fertilizer), as exemplified by agronomic efficiency (Novoa and Loomis, 1981). Others emphasize plant-based efficiency, such as nitrogen recovery efficiency in aboveground biomass (Huggins and Pan, 1993). Still others adopt leaf-based or organ-based perspectives, such as the amount of grain produced per unit of nitrogen accumulated in vegetative organs (e.g., utilization efficiency component). This diversity of definitions, while reflecting different research objectives, can also lead to ambiguity and inconsistency when comparing findings across studies. A more mechanistic approach was proposed by Moll (Moll et al., 1982), who defined NUE as grain yield per unit of nitrogen supplied (from soil and fertilizer) and decomposed it into two components: nitrogen uptake efficiency (NUpE), which reflects the ability of the crop to acquire nitrogen from the soil, and nitrogen utilization efficiency (NUtE), which reflects the efficiency with which the acquired nitrogen is converted into grain yield. Between the two components, NUtE is generally considered more relevant for genetic improvement. This is because high nitrogen accumulation in vegetative tissues does not necessarily translate into high grain yield; rather, the timing and efficiency of nitrogen remobilization from leaves and stems to panicles play a critical role. NUtE directly captures this internal nitrogen translocation process from vegetative organs (leaves and stems) to reproductive organs (panicles), making it a more informative indicator of yield formation and a more suitable target for breeding programs aimed at reducing nitrogen input without compromising productivity.

The conventional methods for assessing NUtE depend heavily on destructive plant sampling and subsequent laboratory-based chemical analysis. These procedures are not only labor-intensive, time-consuming and costly, but also have obvious time lags and operational complexity, which collectively hinder the rapid, large-scale and high-throughput evaluation of NUtE in breeding programs and field production (Chowdhury et al., 2024; Song et al., 2023). In recent years, remote sensing technology has opened up new opportunities for addressing this challenge. In particular, unmanned aerial vehicle (UAV)-based remote sensing offers advantages such as high spatial and temporal resolution, flexibility, and low cost, making it an effective means for rapid and non-destructive monitoring of crop growth status (Liu et al., 2025b; Rejeb et al., 2022). A variety of sensors can be mounted on UAV platforms for crop monitoring. Hyperspectral sensors, while providing rich spectral information, are expensive and generate large data volumes that require complex processing. LiDAR sensors can capture three-dimensional canopy structure but also face high costs and intensive computational demands. In contrast, multispectral sensors offer the advantages of relatively low cost, light weight, and ease of operation, and have been widely used in precision agriculture, making them particularly suitable for routine, high-throughput field phenotyping.

NUtE is essentially a dynamic physiological process that reflects the flow of nitrogen status from vegetative organs to grains during the reproductive stage. This nitrogen translocation drives canopy nitrogen status to change over time throughout the growing season. Since chlorophyll is a nitrogen-containing pigment, changes in canopy nitrogen status generally led to corresponding changes in both chlorophyll content and canopy optical properties. Canopy spectral reflectance is highly sensitive to chlorophyll content, particularly in the visible and red-edge regions. Therefore, by leveraging specific spectral bands (e.g., red or red-edge) that respond sensitively to chlorophyll variation, remote sensing enables the indirect estimation of canopy nitrogen status. Numerous studies have developed vegetation indices (VIs, e.g., NDRE, red-edge chlorophyll index) (Liu et al., 2023; Peng et al., 2024).Thus, by capturing the temporal dynamics of canopy nitrogen via remote sensing, it becomes possible to establish a foundation for estimating NUtE (Gitelson et al., 2022, 2021).

Researchers have proposed various indicators and models using multi-source remote sensing data for assessing crop nitrogen status, including VIs, machine learning algorithms, and radiative transfer model inversion frameworks. For instance, Wang et al. (2021) proposed a novel abundance-adjusted vegetation index to estimate the leaf nitrogen content; Yang et al. (2021) applied convolutional neural networks to retrieve maize leaf nitrogen content from hyperspectral data; Alam et al. (2024) proposed a hybrid inversion framework integrating the PROSAIL radiative transfer model with machine learning algorithms for estimating crop canopy nitrogen. Much of this work has concentrated on nitrogen content rather than NUE. Given that NUE is functionally linked to nitrogen uptake and utilization, several studies have begun to extend this line of research toward NUE estimation. Liu et al. (2022) developed a UAV multispectral-based prediction framework for winter wheat yield and NUE using consumer-grade UAVs; Zhang et al. (2018) demonstrated that photosynthetic nitrogen use efficiency (PNUE) could be assessed using *in situ* hyperspectral remote sensing in winter wheat; and Zhao et al. (2010) developed remote sensing algorithms for estimating nitrogen uptake and NUE in cotton. However, these approaches primarily estimate NUE based on biomass-related efficiencies or organ-level utilization, neither of which captures NUtE from a canopy population perspective. a few studies have targeted the NUtE. Chen et al. (2025) improved maize NUtE estimation by fusing UAV multispectral imagery with LiDAR point cloud data. Qiu et al. (2021) calculated a nitrogen nutrition index and found a significant correlation with measured wheat NUtE. Nevertheless, a physiologically grounded, low-cost, UAV-based multispectral remote sensing approach from a canopy population perspective remains to be developed.

Despite substantial progress, current remote sensing studies have several limitations when it comes to estimating NUtE. Specifically, these limitations are reflected in three aspects: (1) few studies have decomposed NUtE into its physiological components. NUtE integrates two distinct processes: pre-heading nitrogen accumulation in vegetative organs (source formation) and post-heading nitrogen remobilization to grains (sink filling). However, most existing studies treat NUtE as a monolithic target, regressing spectral features directly against the final efficiency value without separating these two processes. (2) Even when multi-temporal spectral data are used, few studies have developed spectral indices or models explicitly designed to target the source-sink transition. As a result, temporal canopy reflectance serves merely as additional input features rather than as direct proxies of the nitrogen remobilization process. (3) the data-driven nature of most existing models constrains their transferability. Models calibrated on specific cultivars or nitrogen rates often fail to generalize to unseen contexts, as they lack grounding in stable physiological mechanisms. In summary, how to effectively link remotely sensed information to the dynamic process of nitrogen translocation underlying NUtE remains a key scientific challenge.

In this study, we aim to develop a physiologically grounded, low-cost, UAV-based multispectral remote sensing approach from a canopy population perspective. Three field experiments were conducted using four rice cultivars with contrasting NUtE and multiple nitrogen application rates. Key agronomic traits and UAV−based multispectral reflectance data were simultaneously acquired at critical growth stages. Specifically, this study has three objectives: (1) to establish a conceptual framework linking rice nitrogen content at key growth stages, which can be easily detected by remote sensing, to rice NUtE; (2) to develop the remote sensing-based method for indicating rice NUtE using spectral indices retrieved from UAV imagery; and (3) to evaluate the effectiveness of the developed approach in rapidly comparing NUtE across different nitrogen treatments and rice cultivars for assisting the selection of high-NUtE cultivars.

## 2 Materials and methods

### 2.1 Study sites and experimental design

Three field experiments were conducted at the Wuhan University Hybrid Rice Experimental Base in Wuhan, Hubei Province (30°33′N, 114°32′E) throughout 2022 to 2024. The study site is characterized by a subtropical monsoon climate, with mean annual temperature of 15.8–17.5 °C and annual precipitation of 1150–1450 mm. Four representative rice cultivars were selected: LY9348 (indica rice), FLY4H (indica rice), CJY582 (japonica rice), and ZH11 (japonica rice). LY9348 possesses high nitrogen utilization efficiency (NUtE), enabling it to maintain relatively high grain yield under low nitrogen supply (Liang et al., 2021b); FLY4H is a high-yielding cultivar with substantial yield potential under sufficient nitrogen conditions but shows moderate NUtE (Xiao et al., 2024); and CJY582 and ZH11, both japonica cultivars, exhibit low to moderately NUtE (Min et al., 2024). The selection of these contrasting cultivars was intended to cover a broad range of nitrogen efficiency types.

Drawing on prior molecular evidence demonstrating that rice exhibits differential responses to varying nitrogen supply levels (Liang et al., 2021b), we designed a gradient of nitrogen fertilizer levels from low to high for each cultivar to characterize the dynamic response of NUtE under varying nitrogen availability. The nitrogen treatments were designed according to local agronomic standards, with nitrogen fertilizer applied in a split pattern: 50% as basal fertilizer 1 day before transplanting, 25% as tillering fertilizer at the early tillering stage, and 25% as panicle fertilizer applied 7–10 days before heading. Notably, the timing of tillering and panicle fertilizer applications was determined based on the specific growth stage of each cultivar, as monitored daily by professional field managers. As summarized in **Table 1**, the experimental design varied by year. In 2022 and 2024, three cultivars (LY9348, FLY4H, and CJY582) were cultivated under three nitrogen regimes (N1/4, N1, N2) with three replications, yielding 27 plots per year. In 2023, an additional cultivar (ZH11) and an extra nitrogen treatment (N0, 0 kg/ha) were incorporated, resulting in a total of four cultivars, four nitrogen levels (N0, N1/4, N1, N2), and three replications, corresponding to 48 plots. Specifically, N1 represents the standard nitrogen application rate (144 kg/ha) for rice production in the region; N1/4 is one-quarter of the standard rate (36 kg/ha); N0 serves as a zero−nitrogen control to assess baseline soil nitrogen supply; and N2 corresponds to twice the standard rate (288 kg/ha) to evaluate crop performance under nitrogen surplus. A total of 270, 384, and 405 samples were collected in 2022, 2023, and 2024, respectively. Multispectral imagery was acquired at key growth stages across all three years. The 2022 and 2023 experiments focused on spectral data collection and grain yield measurement, which informed spectral feature selection and experimental design. The 2024 experiment additionally collected organ biomass, organ nitrogen content, and grain yield. These data were used for model training and validation, while the 2022 and 2023 datasets served as independent external validation sets to evaluate model generalizability across different years.

**Table 1:** Summary of field experiments and sampling information.

Site no.	Year	Location	Plot no.	N treatment	Sample no.
Site 1	2022	Hubei Wuhan	27	N1/4 (36 kg/ha, Low) N1 (144 kg/ha, Standard) N2 (288 kg/ha, High)	270
Site 2	2023	Hubei Wuhan	48	N0 (0 kg/ha, Zero control) N1/4 (36 kg/ha, Low) N1 (144 kg/ha, Standard) N2 (288 kg/ha, High)	384
Site 3	2024	Hubei Wuhan	27	N1/4 (36 kg/ha, Low) N1 (144 kg/ha, Standard) N2 (288 kg/ha, High)	405

### 2.2 Determination of rice NUtE

#### 2.2.1 Definition and rationale

In this study, we focus on NUtE as a core indicator. For rice, nitrogen taken up from the soil is partially allocated to vegetative organs (leaves and stems) during the vegetative stage, and then remobilized to grains (panicle) during the reproductive stage, directly contributing to yield formation. NUtE is commonly defined as the ratio of grain yield to total plant nitrogen accumulation (TPNA) at maturity (Moll et al., 1982), following Equation 1:

(1) $$NUtE=\frac{Yield}{TPNA}$$

Where Grain Yield (kg/ha) is the harvested grain weight determined after threshing, drying, and weighing (as described below), and TPNA is the total nitrogen accumulated in aboveground biomass at harvest, obtained through destructive sampling (as described below). A higher NUtE indicates greater grain yield produced per unit of nitrogen absorbed, reflecting more efficient internal nitrogen utilization and better coordination between nitrogen uptake and remobilization.

#### 2.2.2 Grain yield measurement

At the rice maturity stage, grain yield was determined by randomly selecting three 1 m² quadrats along diagonal lines within each plot. Plants within each quadrat were harvested by cutting at ground level, then bagged and labeled for transport to the laboratory. Following threshing, empty grains and impurities were removed, and the filled grains were oven-dried at 80 °C until constant weight was achieved. The dry grain weight was recorded, and grain yield per unit area was calculated as follows (Equation 2):

(2) $$Yield=\frac{1}{3}\sum_{i=1}^{3}\left(\frac{W_{i}}{1}\times 10\right)$$

Where *Yield* is the grain yield (kg/ha), $W_{i}$ is the dry grain weight of the *i*th quadrat (g/quadrat), the quadrat area is 1 m² in this study, and the factor 10 converts g/m² to kg/ha. The mean value of the three quadrats was taken as the yield for each plot.

#### 2.2.3 Nitrogen distribution and TPNA measurement

Nitrogen distribution among different rice organs exhibits significant variation. Leaves serve as the primary nitrogen storage organ, accumulating substantial nitrogen during vegetative growth to support photosynthesis. Stems act as temporary nitrogen reservoirs, remobilizing nitrogen to grains during the reproductive stage. Panicles, particularly during grain filling, represent the final nitrogen sink, with their nitrogen accumulation directly determining grain yield.

To accurately assess nitrogen status in those organs, destructive sampling was conducted approximately every ten days starting from 10 days after transplanting in each plot, A total of 8 samplings were performed throughout the growing season, covering the tillering, jointing, booting, heading, and maturity stages. At each sampling event, three hills (six plants) were randomly excavated per plot with roots removed. This sampling intensity corresponded to about 6% of the total plants, ensuring sufficient statistical representation while leaving the plots largely undisturbed for subsequent monitoring. Each sampled plant was divided into three fractions: leaves (including blades and sheaths), panicles (after heading, including rachis and grains), and stems. After cleaning to remove soil and debris, each fraction was placed into pre-labeled paper bags. All samples were initially dried at 105 °C for 30 minutes to inactivate biological activity, then dried at 80 °C until reaching a constant weight, and the dry weight of each organ ( Leaf Biomass/ Stem Biomass/ Panicle Biomass) was recorded.

Organ Nitrogen content was determined using the Kjeldahl method (Chowdhury et al., 2024). Dried organ samples were ground with a ball mill, passed through a 0.5 mm sieve, and stored in sealed bags prior to analysis. Approximately 0.5 g (weighed accurately to 0.0001 g) of each sample was placed into a digestion tube, followed by the addition of 5 mL concentrated sulfuric acid (H_2_SO_4_) and 2 g of catalyst mixture (K_2_SO_4_: CuSO_4_·5H_2_O = 10:1, w/w). The mixture was gently mixed and digested on a digestion block at 420 °C for about 90 minutes until the digest became clear and transparent with no visible black particles. After cooling to room temperature, nitrogen concentration was determined using an automatic Kjeldahl analyzer (FOSS Kjeltec 8400, FOSS Analytical AB, Höganäs, Sweden). During distillation, 40% sodium hydroxide (NaOH) solution was added to release ammonia, which was captured by 2% boric acid (H_3_BO_3_) solution. The ammonia absorbed in boric acid was titrated with 0.02 mol/L standard hydrochloric acid (HCl) solution. Nitrogen content was calculated as a percentage on a dry weight basis (as Equation 3):

(3) $$N_{content}=\frac{(V-V_{0})\times C\times 0.014}{m}\times 100\%$$

Where, *V* represents the volume of HCl consumed for sample titration (ml); $V_{0}$represents the volume of HCl consumed for blank titration (ml); *C* represents the concentration of HCl standard solution (mol/L); *m* represents the dry weight of the subsample (g); 0.014 represents the millimolar mass of nitrogen (g/mmol). Nitrogen accumulation in each organ (g/m²) was calculated by multiplying the organ biomass by its corresponding nitrogen concentration:

Nitrogen accumulation in each organ was calculated by multiplying organ dry weight by its corresponding nitrogen content (Equation 4):

(4) $$\begin{array}{l} LNA=Leaf\;Biomass\times N_{leaf\_content}/100\\ SNA=Stem\;Biomass\times N_{stem\_content}/100\\ PNA=Panicle\;Biomass\times N_{panicle\_content}/100 \end{array}$$

Where Leaf Biomass, Stem Biomass, Panicle Biomass represent the biomass of leaves, stems, and panicles, respectively, and LNA, SNA, PNArepresents the nitrogen accumulation in that organ (g/m^2^). TPNA was obtained by summing the nitrogen accumulation of all organs, following Equation 5:

(5) TPNA=LNA+SNA+PNA

Three replicates were analyzed for each sample, and the mean values were used for subsequent data analysis. To standardize data collection across cultivars with different growth durations, we monitored each plot every five days throughout the growing season, with more frequent observations (every 2–3 days) from booting to heading. The heading date of each plot was recorded when 50% of the panicles had emerged. Although all cultivars were sampled on the same calendar dates for practical feasibility, the sampling dates were selected to cover the key phenological stages (e.g., booting, heading, and grain filling) for all cultivars simultaneously. Days to heading (DTH) was then defined as the number of days from heading date to the sampling date, where negative values represent the pre-heading stage and positive values represent the post-heading stage. All subsequent analyses were aligned based on DTH rather than calendar dates, ensuring that comparisons were made at physiologically equivalent time points.

Based on the 2024 experiment, the grain yield and NUtE of the three cultivars are summarized in **Table 2**.

**Table 2:** Grain yield and NUtE of three rice cultivars in 2024.

Cultivar	Grain yield (kg/ha)			NUtE		
	Max	Min	Mean	Max	Min	Mean
FLY4H	7800.06	5047.59	6791.13	0.87	0.48	0.66
CJY582	7480.71	3438.17	5860.29	0.93	0.27	0.56
LY9348	8045.07	6273.65	7516.41	0.99	0.32	0.67

### 2.3 Destructive measurements of canopy pigment contents

Leaf and panicle chlorophyll contents were measured using a spectrophotometric extraction method. For each plot, the topmost fully expanded leaf and the main panicle (after heading) were collected from three sampled rice hills and transported to the laboratory. A handheld leaf punch (Corrida Technology Co., Ltd., Zhuhai, China) was used to collect 6 mm diameter discs for leaf samples. For panicle samples, 2–3 mm fragments were prepared using sterile scissors. Fresh tissue (0.1 g) was weighed and placed in pre-cooled mortars, then thoroughly ground with 5 mL of 95% ethanol (analytical grade) in an ice bath until complete disruption was achieved. The solution was transferred to a volumetric flask and diluted with ethanol to a final volume of 10 mL, followed by centrifugation at 4000 rpm for 10 minutes at 4 °C. The supernatant was filtered through a 0.22 μm organic membrane filter prior to spectrophotometric analysis. Absorbance was measured at 664 nm and 647 nm using a UV-VIS spectrophotometer (Aoe Instruments Co., Ltd., Shanghai, China). Chlorophyll concentrations were calculated according to Lichtenthaler’s equations (Lichtenthaler, 1987). Chlorophyll content per unit fresh weight (mg/g) was calculated as Equation 6:

(6) $$Chl(mg/g)=\frac{1000\times C\times V_{total}}{FW}$$

Where *C* is the chlorophyll concentration (mg/L), $V_{total}$ is the final volume (10 mL), and *FW* is the fresh weight (0.1 g).

It should be noted that while leaf chlorophyll content was initially measured on a fresh weight basis (mg/g FW) for immediate laboratory analysis, the canopy-level chlorophyll content (CCC, mg/m) was subsequently calculated using biomass on a dry weight basis (g/m), following Equation 7. This approach was adopted because rice has inherently high and variable leaf water content (typically 85–90%), and fresh weight is subject to diurnal fluctuations due to transpiration as well as water loss during post-sampling handling (Haefele et al., 2010; Yang et al., 2024). Using dry weight for biomass normalization ensures greater stability and comparability across samples.

(7) $$CCC=Chl_{leaf}\times Leaf\;Biomass+Chl_{panicle}\times Panicle\;Biomass$$

Where $Chl_{leaf}$ and $Chl_{panicle}$ are the chlorophyll contents of leaves and panicles (mg/g), respectively, and Leaf Biomass and Panicle Biomass are the leaf biomass and panicle biomass (g/m²), respectively, obtained from the destructive sampling described in Section 2.2.

### 2.4 UAV imaging and canopy reflectance

Multispectral images for the study sites were acquired using an M8 UAV (TT Aviation Technology Co., Ltd., Beijing) equipped with a Mini-MCA multispectral camera (Tetracam Inc., Chatsworth, CA, USA), which operated at center bands of 490, 520, 550, 570, 670, 680, 700, 720, 800, 850, 900, and 950 nm. Prior to flight, the camera system was calibrated in the laboratory to ensure that corresponding pixels of each lens were spatially overlapping in the same focal plane. During flight, a gimbal stable platform was used to adjust the camera system pointing close to nadir. For each exposure, images of twelve bands were simultaneously obtained over the study sites. Flights were conducted under clear sky conditions around 11:30 am local time, when changes in the solar zenith angle were minimal. The flight altitude was maintained at approximately 100 m, and the ground resolution was 5.5 cm per pixel. At this altitude, the images fully covered the experimental field.

Image preprocessing involved two steps: geometric correction (band registration) and radiometric calibration. First, band registration was performed using PixelWrench2 software (Tetracam Inc., Chatsworth, CA, USA) to align all spectral bands for each image. Second, radiometric calibration was applied to convert digital numbers (DN) to reflectance values using constant reflectance targets. Specifically, the sub-band empirical line method was adopted. Eight reference panels with known reflectance values (0.03, 0.06, 0.12, 0.24, 0.36, 0.48, 0.56, and 0.80) were placed at the edge of the field to ensure they were captured in the same images as the experimental plots. For each spectral band, the relationship between the DN of the reference panels and their known reflectance values was modeled using a linear regression (Equation 8):

(8) R=a×DN+b

Where *R* is the reflectance, *DN* is the digital number recorded by the sensor, and *a*(gain) and *b*(offset) are the calibration coefficients. The gain and offset for each band were derived using the least squares method by minimizing the sum of squared residuals between the measured *DN* and the known reflectance values (Equation 9):

(9) $$\min\sum_{i=1}^{n}{\left(R_{i}-(a\times DN_{i}+b)\right)}^{2}$$

Where n=8 represents the number of reference panels. The derived calibration coefficients were then applied to all pixels within the images to convert *DN* to reflectance.

For each plot, a region of interest (ROI) was defined to achieve the maximal intersection with the outline of the observation zone of the plot. The pixels for plants at the edge two lines were excluded from the ROI, since those edge plants obviously grew denser and higher due to much less intraspecific resource competition. The remaining pixels were considered to represent plants of the same cultivar under similar environmental and resource competition conditions. Since this study focused on the plant stand of a specific cultivar, the canopy reflectance for that rice cultivar was the plot-level reflectance retrieved by averaging all pixel values within the ROI. Subsequently, the average reflectance of each plot was obtained for further analysis.

## 3 Methodology of developing the remote indicator for rice NUtE

### 3.1 The conceptual framework of linking NUtE to rice nitrogen content

**Figure 1:**
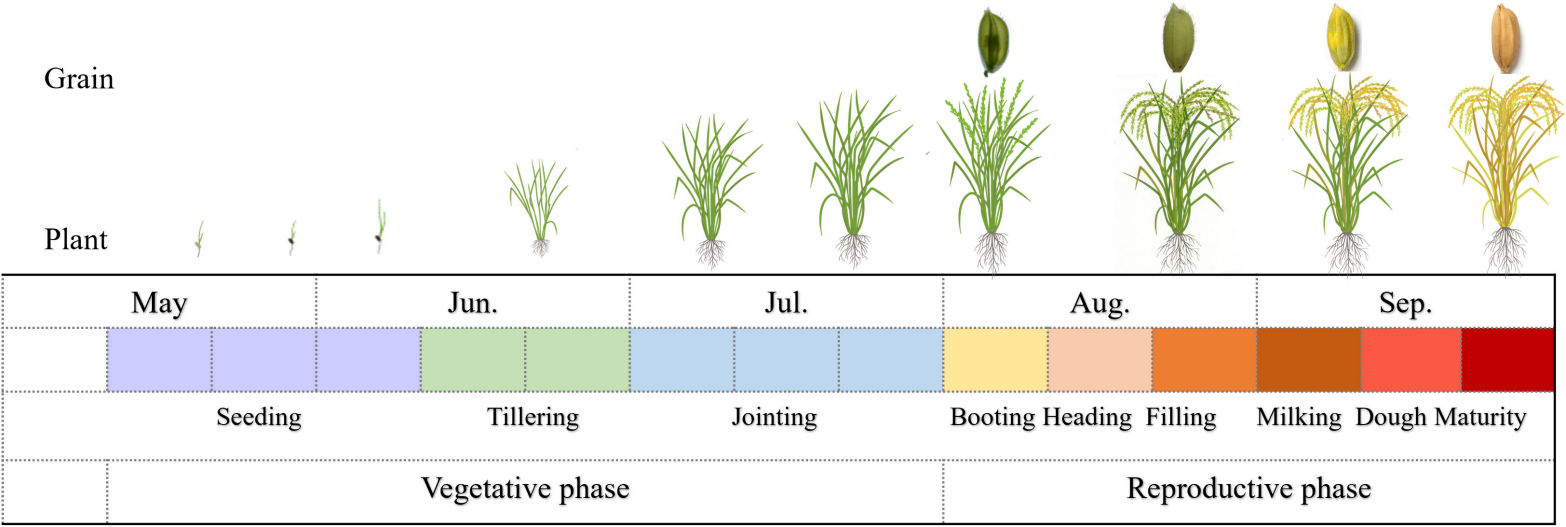
Schematic diagram of rice growth stages and grain development.

The rice growing season at our study site extends from May to late September, encompassing two principal developmental phases: the vegetative phase and the reproductive phase (**Figure 1**). The vegetative phase comprises the seeding, tillering, and jointing stages. During this phase, the plant undergoes root system establishment, stem elongation, and leaf area expansion, thereby constructing the morphological architecture necessary for subsequent growth and development. Photosynthetic assimilates are preferentially allocated to vegetative organs, while nitrogen uptake remains highly active, with the majority of absorbed nitrogen being stored in leaves and stems as a transient reserve pool. The reproductive phase commences with booting, followed sequentially by heading, filling, milking, dough, and finally maturity. Following heading, the panicle emerges and grain initiation occurs. The plant transitions from vegetative growth to grain development, during which stored reserves are progressively remobilized from senescing leaves and stems toward the developing panicles. This transitional window represents a critical period for nitrogen remobilization, as the efficiency of nutrient translocation during this phase exerts a direct influence on ultimate grain yield formation.

Nitrogen accumulation and partitioning in rice follow distinct temporal patterns. During the vegetative phase, nitrogen is primarily accumulated in leaves and stems, forming a nitrogen pool that serves as the source for grain development. After heading, this stored nitrogen is remobilized to panicles, where it accumulates as the sink that ultimately determines grain yield (Guo et al., 2025b; Shang et al., 2024; Xu et al., 2024). This clear separation between source establishment and sink filling suggests that NUtE can be decomposed into two distinct but interrelated components: the nitrogen pool accumulated during vegetative phase (source) and the nitrogen remobilized to panicles during post-heading stage (sink).

In rice, the total plant nitrogen pool is established predominantly during the vegetative phase. From tillering to booting, nitrogen taken up by roots is preferentially allocated to vegetative organs such as leaves and stems. These organs serve as nitrogen storage pools, utilizing part of the nitrogen for their own growth and development while storing the surplus in forms such as proteins and chlorophyll within cells. As the growing season progresses, nitrogen accumulation in leaves and stems increases continuously, reaching its peak at booting (Liu et al., 2025a). This nitrogen pool constitutes the primary source for subsequent grain filling, and its magnitude directly determines the availability of nitrogen for remobilization during the reproductive phase (Kunwar et al., 2025). From a physiological perspective, nitrogen accumulation in vegetative organs reflects the overall nitrogen status of the plant. On one hand, leaf nitrogen content directly affects photosynthetic capacity and assimilate production; on the other hand, the nitrogen stored in leaves and stems serves as the main source for remobilization to panicles during grain filling, and its quantity determines the potential nitrogen supply for grain development. Therefore, the indicator for the total plant nitrogen pool should be selected from nitrogen accumulation in vegetative organs during the vegetative phase. Accordingly, TPNA at maturity is primarily determined by nitrogen accumulation in vegetative organs (leaves and stems) during the vegetative stage (Equation 10):

(10) $$TPNA\propto N_{\text{vegetative}}$$

Where $N_{\text{vegetative}}$ is the nitrogen accumulation in vegetative organs (leaves and stems) during the vegetative phase (e.g., booting), representing the total plant nitrogen pool (source). In this study, we analyzed the relationships between TPNA at maturity and nitrogen accumulation in leaves and stems at key vegetative stages, including tillering, early jointing, late jointing, and booting.

Grain yield is an accumulated outcome primarily determined by the continuous remobilization and accumulation of nitrogen in panicles during grain filling. After heading, the physiological function of vegetative organs shifts: nitrogen stored in leaves and stems begins to degrade and is exported, transported via the phloem to the panicles, where it is incorporated into grain proteins, driving grain filling and kernel development (Li et al., 2025). This process is not instantaneous but extends throughout the grain filling period, with nitrogen remobilization rates varying across post−heading stages and panicle nitrogen accumulation increasing progressively. From a physiological perspective, the panicle serves as the terminal nitrogen sink (Hu et al., 2025). Its nitrogen accumulation reflects both the extent of grain filling and the final yield level. The efficiency of nitrogen remobilization directly affects protein accumulation in grains and grain plumpness. Therefore, the indicator for nitrogen remobilization capacity should be selected from panicle nitrogen accumulation increments across post−heading stages. Accordingly, grain yield is primarily determined by the increment in panicle nitrogen accumulation from heading to grain filling (Equation 11):

(11) $$Yield\propto \text{Δ}N_{\text{panicle}}$$

Where $\text{Δ}N_{\text{panicle}}$denotes the increment in panicle nitrogen accumulation from heading ( $N_{panicle,heading}$) to a given post-heading stage ( $N_{panicle,post-heading}$), calculated as the difference between panicle nitrogen at that stage and that at heading, representing the amount of nitrogen remobilized to panicles (sink). In this study, we tested the correlations between panicle nitrogen accumulation at maturity and the incremental nitrogen accumulation in leaves, stems, and panicles during successive post-heading intervals, specifically from heading to filling, heading to milking, heading to dough, and heading to maturity.

Therefore, together with the definition of NUtE from Equation 9, we made the following assumptions linking NUtE to organ-specific nitrogen accumulation and its temporal dynamics in rice.

(12) $$NUtE\propto \frac{\text{Δ}N_{\text{panicle}}}{N_{\text{vegetative}}}$$

where $N_{\text{vegetative}}$ is the nitrogen accumulation in vegetative organs (leaves and stems) during the vegetative stage, representing the total plant nitrogen pool (source); $\Delta N_{\text{panicle}}$ and is the increment in panicle nitrogen accumulation from heading to grain filling, representing the amount of nitrogen remobilized to panicles (sink).

### 3.2 Remote estimation of rice organ nitrogen content as a proxy of NUtE

As established in the previous section, NUtE is defined as the ratio of grain yield to TPNA at maturity and can be decomposed into source and sink components. Nitrogen accumulation and remobilization are inherently dynamic: nitrogen is first stored in vegetative organs and then translocated to panicles after heading. Capturing the full temporal dynamics of organ-specific nitrogen content from the vegetative phase through maturity is therefore a prerequisite for understanding and estimating NUtE. Equation 12 provides a quantitative linkage between NUtE and organ nitrogen content, indicating that NUtE can be derived from organ-specific nitrogen accumulation measured at key growth stages. This implies that accurate quantification of nitrogen content in leaves, stems, and panicles across multiple stages is fundamental for reliable NUtE estimation. Remote sensing offers a feasible, non-destructive approach for such quantification, yet its estimation accuracy directly determines the reliability of NUtE retrieval. Accordingly, this study aims to explore the potential of multispectral remote sensing for estimating nitrogen content in rice leaves, stems, and panicles at critical growth stages, with the goal of developing a remote sensing-based framework for NUtE assessment.

First step is to understand the spectral basis for estimating nitrogen content from remote sensing data. Nitrogen is a core constituent of chlorophyll molecules, with approximately 50-70% of canopy nitrogen invested in photosynthetic apparatus, including chlorophyll proteins and Rubisco (Guo et al., 2025a; Onoda et al., 2017). A strong positive correlation has been established between canopy nitrogen content and chlorophyll concentration across diverse crop species, including rice (Fu et al., 2021; Inoue et al., 2012; Li et al., 2022). This close relationship arises because nitrogen availability directly regulates chlorophyll synthesis and chloroplast development. Consequently, canopy chlorophyll content serves as an effective proxy for canopy nitrogen status, enabling the estimation of canopy nitrogen content through the retrieval of chlorophyll-related spectral information.

The retrieval of chlorophyll-related spectral information, in turn, relies on the optical properties of chlorophyll pigments. The feasibility of estimating chlorophyll content from remote sensing data is fundamentally governed by the distinctive spectral properties of chlorophyll pigments. Chlorophyll exhibits strong absorption in the visible region, particularly in the red and blue bands, while reflecting strongly in the near-infrared (NIR) region due to leaf internal structure (Gitelson and Merzlyak, 1998; Jacquemoud and Ustin, 2001). This sharp contrast between low reflectance in the red and high reflectance in the NIR forms the biophysical basis of vegetation indices, such as the Normalized Difference Vegetation Index (NDVI) (Rouse et al., 1973). Moreover, the red edge region, where reflectance increases dramatically from red to NIR, is particularly sensitive to chlorophyll concentration variations (Gitelson et al., 2005, 1996; Gitelson and Merzlyak, 1998). As chlorophyll content decreases, the red edge shifts toward shorter wavelengths (blue shift), whereas high chlorophyll content causes a red edge shift toward longer wavelengths (red shift). These spectral features allow quantitative retrieval of chlorophyll content using empirically calibrated vegetation indices or physically based radiative transfer models.

Therefore, in this study, nine chlorophyll-related vegetation indices (Chl−related VIs) and one nitrogen-related VI were calculated using the plot-level canopy reflectance obtained in Section 2.4 (see **Table 3**). In addition, days to heading (DTH), as defined in Section 2.2, was included as a predictor variable to account for phenological differences among cultivars. These predictors were then used to estimate source ( $N_{\text{vegetative}}$) and sink ( $\text{Δ}N_{\text{panicle}}$) via regression and machine learning methods, as described below.

**Table 3:** The summary of vegetation indices (VI) used in this study.

VI	Formula	Reference
Normalized difference vegetation index (NDVI)	$\left(R_{800}-R_{670})/(R_{800}+R_{670}\right)$	(Rouse et al., 1973)
Enhanced vegetation index 2 (EVI2)	$2.5*(R_{800}-R_{670})/(R_{800}+2.4*R_{670}+1)$	(Birth and McVey, 1968)
Renormalized difference vegetation index (RDVI)	$(R_{800}-R_{670})/\sqrt{\left(R_{800}+R_{670}\right)}$	(Roujean and Breon, 1995)
Ratio vegetation index (RVI)	$R_{800}/R_{670}$	(Jordan, 1969)
Normalized difference red edge index (NDRE)	$\left(R_{800}-R_{720})/(R_{800}+R_{720}\right)$	(Gitelson and Merzlyak, 1994)
Red edge chlorophyll index (CI_red edge_)	$R_{800}/R_{720}-1$	(Gitelson et al., 2003)
MERIS terrestrial chlorophyll index (MTCI)	$\left(R_{800}-R_{720})/(R_{720}+R_{670}\right)$	(Dash and Curran, 2004)
Green normalized difference vegetation index (GNDVI)	$\left(R_{800}-R_{550})/(R_{800}+R_{550}\right)$	(Gitelson et al., 1996)
Green chlorophyll index (CI_green_)	$R_{800}/R_{550}-1$	(Gitelson et al., 2003)
Double-peak canopy nitrogen index (DCNI)	$\frac{1/R_{720}-1/R_{700}}{1/R_{720}-1/R_{670}}\times \frac{1}{R_{720}-R_{670}+0.03}$	(Chen et al., 2010)

In this study, linear regression models were first employed. Simple regression was used to establish linear relationships between nitrogen accumulation and individual predictors. Multiple regression with stepwise selection was then applied to identify the most significant predictors among multiple vegetation indices for estimating nitrogen accumulation. To capture potential nonlinear relationships that linear models may fail to address, three machine learning algorithms were also employed: Multilayer Perceptron (MLP), which captures nonlinear relationships through multiple hidden layers; K-Nearest Neighbors (KNN), which estimates nitrogen accumulation based on the average of the k most similar samples in the feature space; and Gradient Boosting (GB), which sequentially builds multiple decision trees, with each new tree trained to correct errors from previous ones. All methods were executed using the scikit-learn package in Python 3.8.

Upon achieving reliable remote estimation of organ-specific nitrogen content, NUtE can be indirectly derived based on its source-sink decomposition framework. Accordingly, this study characterizes NUtE by indirectly estimating vegetative organ nitrogen accumulation during the pre-heading vegetative stage (source) and post-heading panicle nitrogen increment (sink). Following this rationale, we constructed a fully remotely sensible index (Equation 13), designated the Nitrogen Utilization Efficiency-Vegetation Index (NUtE-VI), as a remote sensing proxy for NUtE:

(13) $$NUtE\_VI=\frac{f(\sin k_{VI})}{f(Sourse_{VI})}$$

where $Sourse_{VI}$denotes the remotely estimated leaf nitrogen accumulation during the pre-heading stage, serving as a proxy of $N_{\text{vegetative}}$ in Equation 12 and $\sin k_{VI}$denotes the remotely estimated panicle nitrogen accumulation increment from heading to maturity, serving as a proxy of $\text{Δ}N_{\text{panicle}}$ in Equation 12. The functional form of *f* was determined using the regression and machine learning methods described above, with field-measured NUtE serving as the calibration target, thereby capturing both the establishment of the vegetative nitrogen pool and its subsequent remobilization to grains, and serves as a spectrally based indicator of NUtE in rice.

Prior to model training, we cleaned the data by removing samples with missing values and excluding extreme outliers (values beyond three standard deviations from the mean). Multi-year experimental data collected from 2022 to 2024 were used for model development and validation. A total of 405 samples were collected in 2024, which were randomly split into a training set of 283 samples (70%) for model calibration and a validation set of 122 samples (30%) for hyperparameter tuning. The trained models were then directly applied to the independent test datasets from 2022 (270 samples) and 2023 (384 samples) to assess their generalizability across different years, cultivars, and nitrogen treatments.

**Figure 2:**
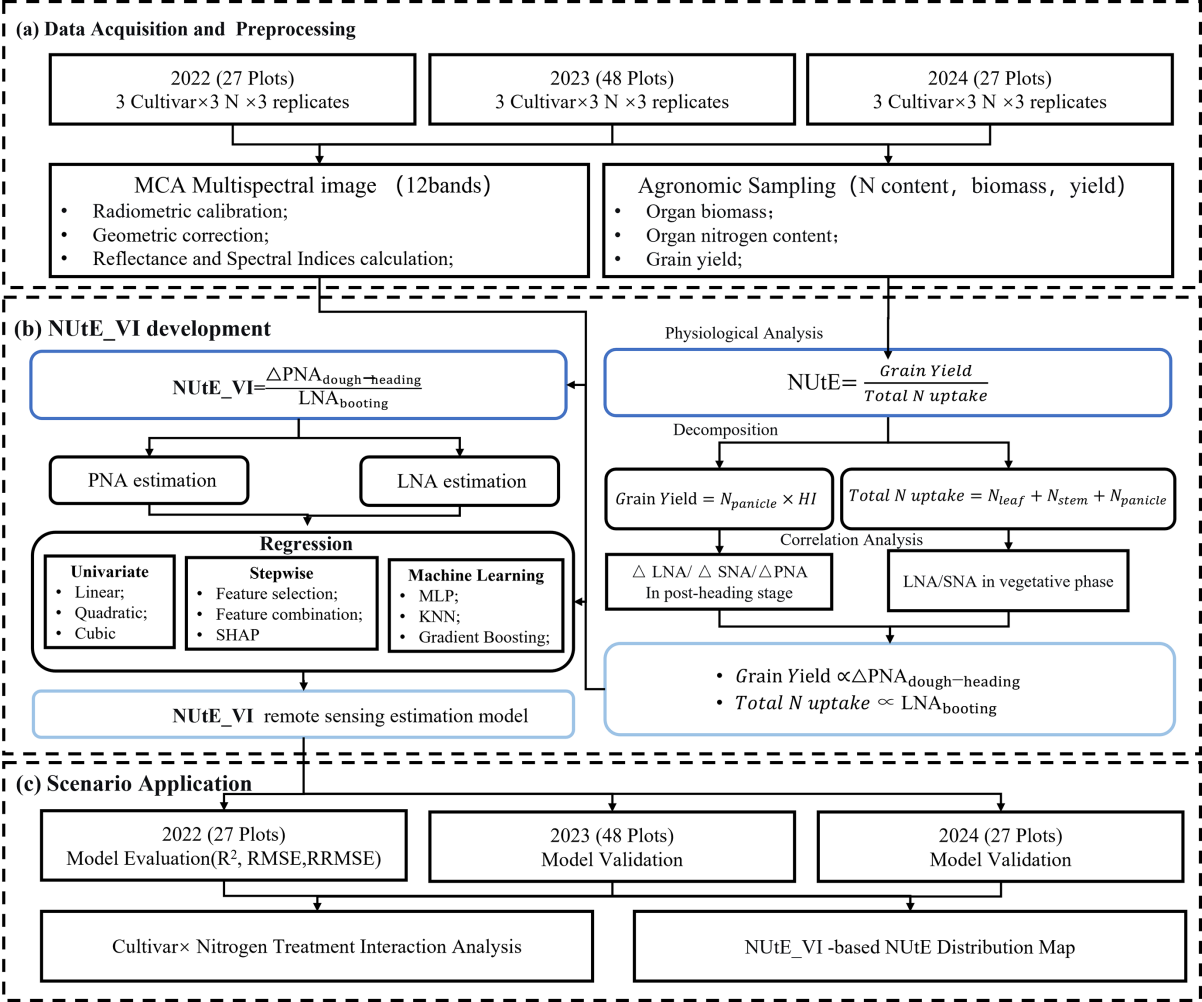
The technical workflow of this study. **(A)** Data acquisition and preprocessing from field experiments (2022–2024). **(B)** Development of NUtE-VI using regression and machine learning methods. **(C)** Model validation, interaction analysis, and NUtE mapping.

For model training, hyperparameters for each machine learning algorithm were optimized via grid search combined with 5-fold cross-validation on the training set, using RMSE as the evaluation metric. The optimal hyperparameter combination was selected based on the lowest cross-validated RMSE, and the final model was retrained on the full training set using the selected hyperparameters. The technical workflow is illustrated in **Figure 2**.

Four statistical indicators were used to evaluate model performance (Equations 14–17): the Pearson correlation coefficient (r), coefficient of determination (R²), root mean square error (RMSE), and relative root mean square error (rRMSE). For r and R², values closer to 1 indicate a stronger linear correlation between predicted and observed values and higher explanatory power. For RMSE and rRMSE, smaller values indicate lower prediction errors and higher estimation accuracy. The formulas for these indicators are as follows:

(14) $$r=\frac{\sum \left(x_{i}-\bar{x})(y_{i}-\bar{y}\right)}{\sqrt{\sum {\left(x_{i}-\bar{x}\right)}^{2}}\sqrt{\sum {\left(y_{i}-\bar{y}\right)}^{2}}}$$

(15) $$R^{2}=1-\frac{\sum {\left(x_{i}-y_{i}\right)}^{2}}{\sum {\left(x_{i}-\bar{x}\right)}^{2}}$$

(16) $$RMSE=\sqrt{\frac{\sum {\left(x_{i}-y_{i}\right)}^{2}}{n}}$$

(17) $$rRMSE=\frac{RMSE}{\bar{y}}$$

Where $x_{i}$ and $y_{i}$ are the observed and predicted values, respectively, $\bar{x}$ and $\bar{y}$ are the means of the observed and predicted values, ${\hat{y}}_{i}$ is the model-predicted value, and *n* is the number of samples.

## 4 Result

### 4.1 Developing the linkage of NUtE and rice nitrogen content at key growth stages

**Figure 3:**
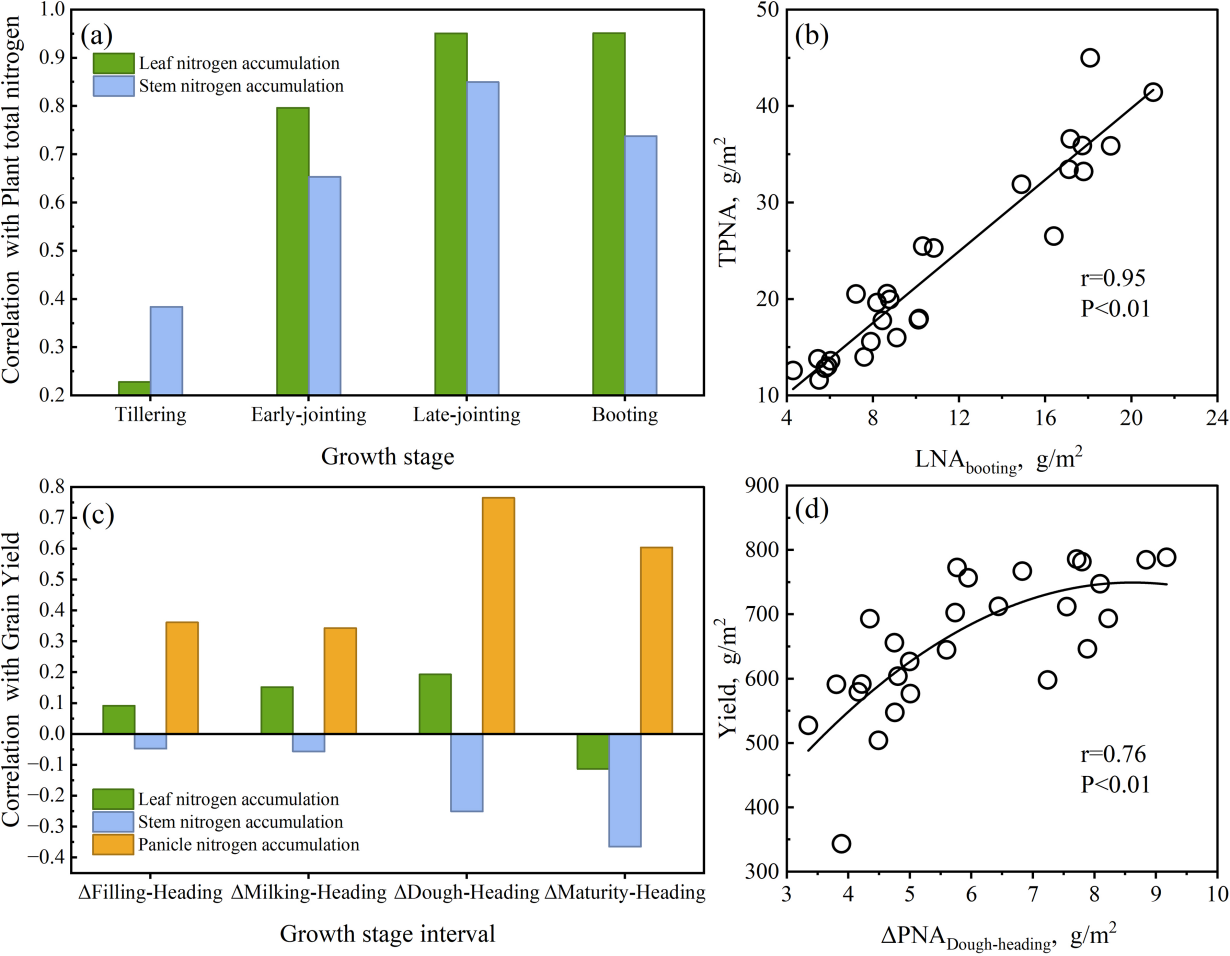
Relationships between **(A)** leaf and stem nitrogen accumulations at four vegetative growth stages and total plant nitrogen accumulation (TPNA) at maturity; **(B)** leaf nitrogen accumulation at booting stage (LNA_booting_) and total plant nitrogen accumulation (TPNA); **(C)** nitrogen accumulation increments in leaves, stems, and panicles during four post−heading intervals and grain yield; **(D)** panicle nitrogen accumulation from heading to dough (ΔPNA_dough-heading_) and grain yield. All correlation analyses were based on 27 plot-level samples (n = 27).

To identify key physiological indicators for NUtE-VI, we analyzed the relationships between organ−specific nitrogen dynamics and total plant nitrogen accumulation (TPNA), grain yield at maturity (**Figure 3**). Four vegetative growth stages (tillering, early jointing, late jointing, and booting) were considered for TPNA, and four post−heading intervals (heading to filling, heading to milk, heading to dough, heading to maturity) for grain yield.

For TPNA at maturity, the correlations between leaf and stem nitrogen accumulations at four vegetative growth stages and TPNA are shown in **Figure 3A**. Leaf nitrogen accumulation (LNA) showed a rapidly increasing positive correlation from tillering (r = 0.23) to late jointing (r = 0.94), with only a slight increase from late jointing to booting (r = 0.94 to 0.95). Stem nitrogen concentration (SNA) peaked at late jointing (r = 0.85). These results indicate that both leaves and stems function as nitrogen storage pools during vegetative phase, with leaves playing a dominant role. Among all stages, LNA at booting stage (LNA_booting_) exhibited the highest correlation with TPNA (r = 0.95, p < 0.01), as illustrated in **Figure 3B**.

For grain yield, the correlations between nitrogen changes in leaves, stems, and panicles at four post−heading growth stages and grain yield are presented in **Figure 3C**. Leaf nitrogen accumulation increments (ΔLNA) showed a weak positive correlation at heading to filling (r = 0.09) and turned negative thereafter (r = -0.01 to -0.30), reflecting the onset of nitrogen export from leaves. Stem nitrogen accumulation increments (ΔSNA) were consistently negatively correlated across all post−heading intervals (r = -0.05 to -0.45), indicating continuous nitrogen remobilization from stems after heading. In contrast, panicle nitrogen accumulation increments (ΔPNA) were positively correlated with yield throughout the post−heading stage, with the correlation increasing from heading to filling (r = 0.36) to heading to dough (r = 0.76), before declining slightly at later stages. The relationship between panicle nitrogen accumulation from heading to dough (ΔPNA_dough-heading_) and grain yield showed highest correlation with grain yield (r = 0.76, p < 0.01; **Figure 3D**).

Based on these two indicators, the NUtE-VI was constructed as the ratio of ΔPNA_dough-heading_ to LNA_booting_ (Equation 18).

(18) $$NUtE\_VI=\frac{PNA_{dough-heading}}{LNA_{booting}}$$

**Figure 4:**
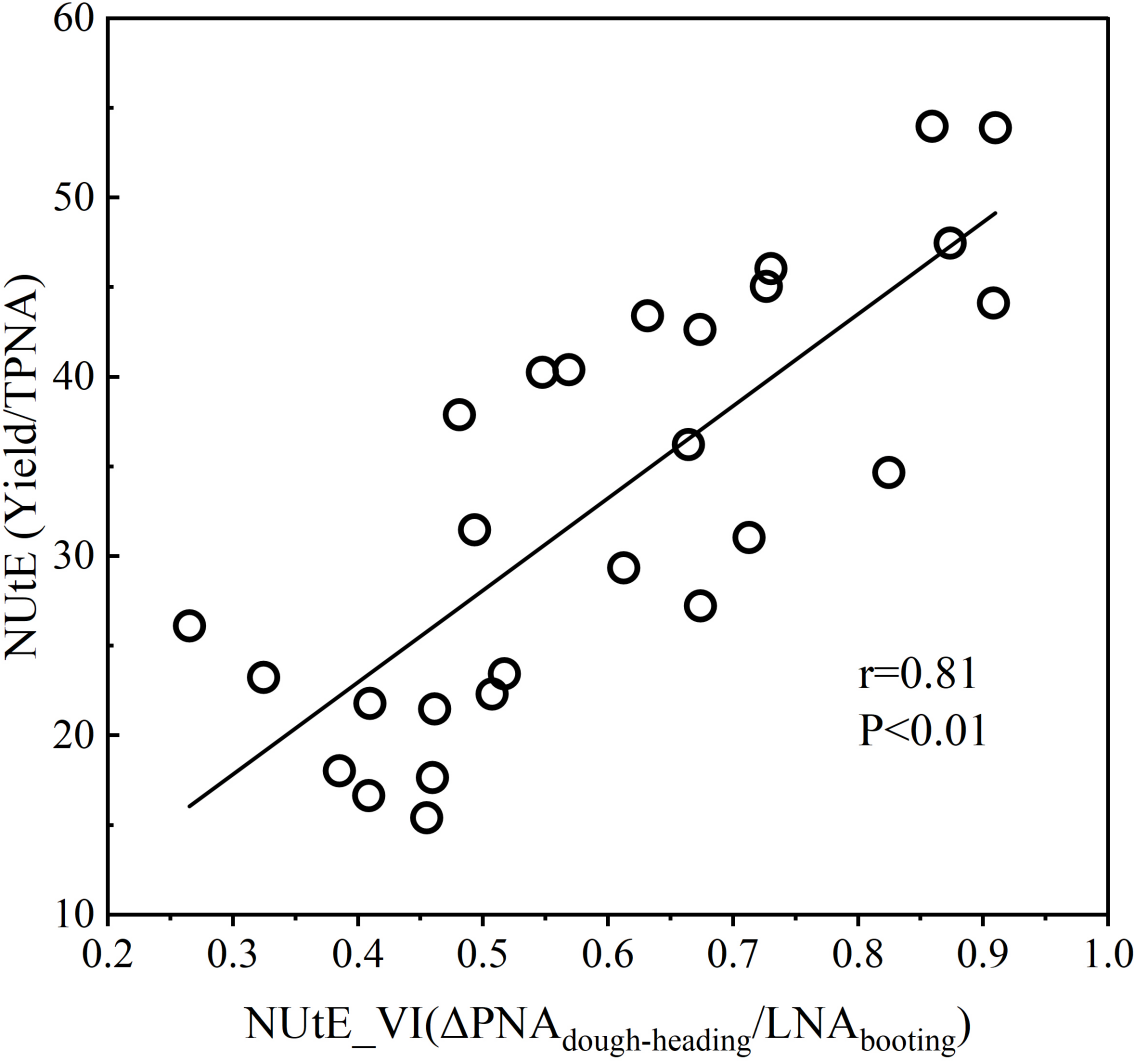
Scatters between the NUtE_VI (ΔPNA_dough-heading_/LNA_booting_) and nitrogen utilization efficiency (yield/TPNA).

Where $PNA_{dough-heading}$ is panicle nitrogen accumulation from heading to dough stage, and $LNA_{booting}$ is leaf nitrogen accumulation at booting stage. The relationship between NUtE-VI and NUtE (Yield/TPNA) is shown in **Figure 4**, with a significant positive correlation (r = 0.81, p < 0.01), confirming its potential as an effective physiological proxy for NUtE.

### 4.2 Remote estimation of leaf nitrogen content and panicle nitrogen content

**Figure 5:**
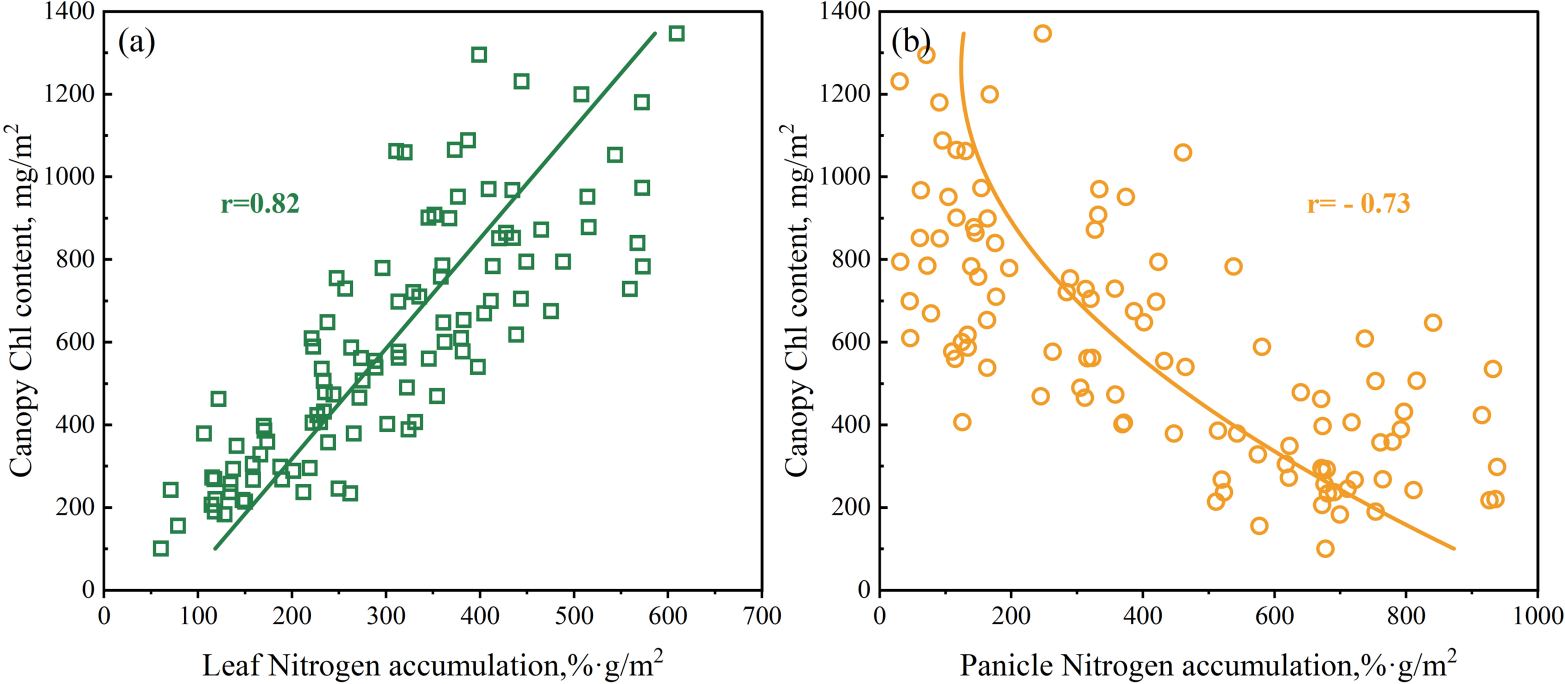
Relationships between canopy chlorophyll content (CCC) and **(A)** leaf nitrogen accumulation (LNA) and **(B)** panicle nitrogen accumulation (PNA) in rice.

**Figure 5** illustrates the relationships between canopy chlorophyll content (CCC) and LNA, PNA. CCC exhibited a significant positive correlation with LNA (r = 0.82, p < 0.01; **Figure 5A**), consistent with the fact that canopy chlorophyll is primarily located in leaves and reflects leaf nitrogen status. In contrast, **Figure 5B** shows a significant negative correlation between CCC and PNA (r = -0.73, p < 0.01), with the decline rate of CCC accelerating as PNA increased. This negative relationship is associated with the grain filling process, during which nitrogen progressively accumulates in panicles while leaf chlorophyll degrades, leading to a concurrent decrease in CCC.

**Figure 6:**
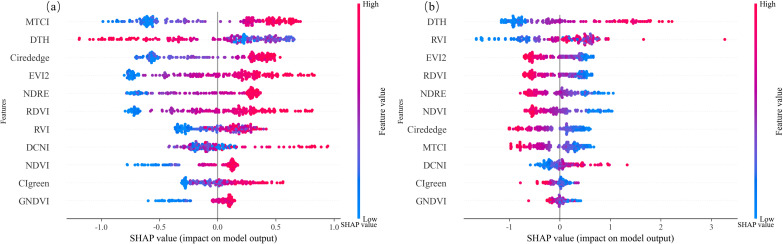
SHAP summary plots for **(A)** leaf nitrogen accumulation (LNA) and **(B)** panicle nitrogen accumulation (PNA) estimation.

To further evaluate the contribution of different vegetation indices (VI) to the estimation of LNA and PNA, SHAP analysis was performed on the optimal machine learning models, with results shown in **Figure 6A** (LNA) and **Figure 6B** (PNA). The features are ranked by importance from top to bottom, with the color representing the feature value (red: high, blue: low) and the horizontal position indicating the SHAP value, reflecting the direction and magnitude of each feature’s contribution to the model output. For LNA estimation (**Figure 6A**), Chl-related VIs dominated the feature importance ranking and consistently exhibited positive contributions, indicating that higher values of these indices were associated with higher predicted LNA. MTCI ranked first, with the highest positive SHAP values, Days to heading (DTH) ranked second but showed a negative contribution, which is consistent with its definition. With the exception of a few samples, DCNI exhibited a negative contribution, and its SHAP value ranked relatively low among all features. The remaining indices also exhibited positive contributions, though with relatively lower SHAP values. The consistent positive contributions of Chl-related VIs align with the physiological relationship between leaf chlorophyll and nitrogen content, confirming their effectiveness for estimating LNA.

For PNA estimation (**Figure 6B**), DTH ranked first with a distinctly positive contribution, reflecting that the progression of grain filling (increasing DTH) is closely associated with PNA. In contrast, most Chl-related VIs exhibited negative contributions. Among them, RVI ranked second, followed by EVI2 in third position, both showing negative contributions. DCNI were the only index that showed positive contributions, though with relatively lower SHAP values. The predominance of negative contributions among Chl-related VIs aligns with the negative correlation between canopy chlorophyll content and panicle nitrogen accumulation observed in **Figure 5B**, reflecting the nitrogen remobilization process during grain filling, during which leaf chlorophyll degrades while nitrogen accumulates in panicles.

**Figure 7:**
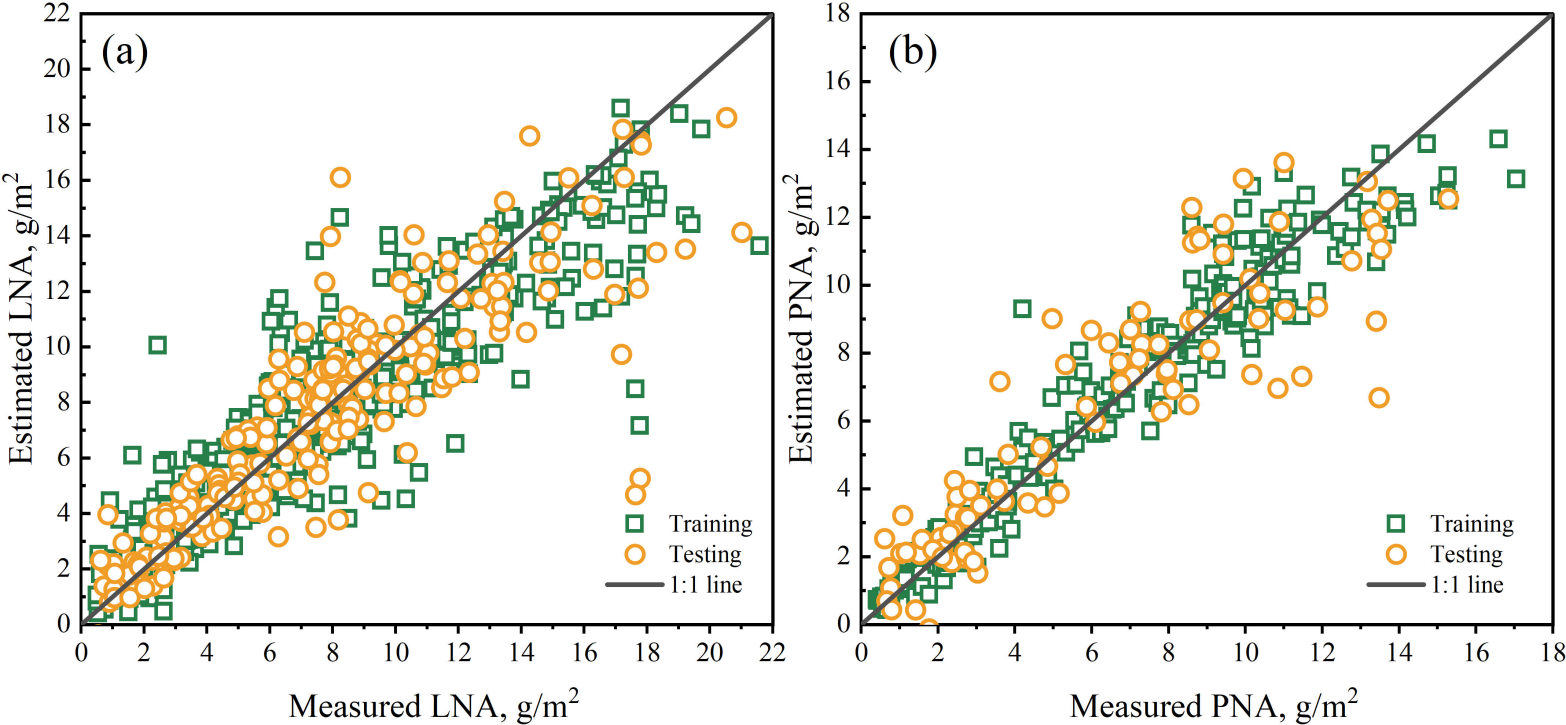
Scatter plots of measured vs. estimated **(A)** leaf nitrogen accumulation (LNA) and **(B)** panicle nitrogen accumulation (PNA) using the best-performing MLP model.

Based on the SHAP analysis, the top seven features that collectively accounted for over 90% of the total contribution were selected as predictors for model development. The models based on different predictors for estimating rice PNA and LNA were developed and compared (**Table 4**). For LNA estimation, among the simple regression models, the performance of various VIs and DTH was comparable, with testing R² ranging from 0.27 to 0.62. EVI2 showed relatively better performance (testing R² = 0.62). Multiple stepwise regression improved testing R^2^ to 0.73 with an RMSE of 2.50 g/m², indicating that multivariate combinations outperform single predictors for LNA estimation. Machine learning models demonstrated clear advantages. Compared to the best simple regression model (EVI2), MLP reduced RMSE by 51.4% (from 3.27 to 1.59 g/m²) and rRMSE by 67.5% (from 26.83% to 8.71%) on the testing set. Overall, testing R^2^ for machine learning models reached 0.85–0.89, with RMSE decreasing to 1.59–1.89 g/m² and rRMSE ranging from 8.7% to 11.3%. Among them, MLP performed best, achieving a testing R^2^ of 0.89 and an rRMSE of 8.71% (**Figure 7A**).

**Table 4:** Performance of different models for estimating leaf nitrogen accumulation (LNA) and panicle nitrogen accumulation (PNA).

Target	Regression	VI	Training			Testing		
			R^2^	RMSE g/m^2^	rRMSE %	R^2^	RMSE g/m^2^	rRMSE %
Leaf nitrogen accumulation	Simple regression	RDVI	0.56	3.23	24.36	0.45	3.74	28.03
		EVI2	0.54	3.31	25.87	0.62	3.27	26.83
		CI_red edge_	0.47	3.60	34.76	0.51	3.50	34.50
		DTH	0.53	3.63	37.50	0.54	3.95	40.86
	Multiple regression	All VI	0.54	3.06	18.96	0.73	2.50	15.92
	MLP	7 VIs^*^	**0.85**	**1.73**	**9.30**	**0.89**	**1.59**	**8.71**
	KNN		0.81	1.95	10.86	0.85	1.87	10.44
	GB		0.89	1.48	7.40	0.86	1.89	11.30
Panicle nitrogen accumulation	Simple regression	DTH	0.81	1.88	13.15	0.76	2.50	20.32
		NDRE	0.39	3.40	38.38	0.23	3.59	40.77
		MTCI	0.39	3.40	38.38	0.23	3.59	40.77
		CI_red edge_	0.29	3.46	35.95	0.05	3.69	40.27
	Multiple regression	All VI	0.85	1.66	11.29	0.76	2.37	18.74
	MLP	7 VIs^*^	**0.95**	**1.06**	**7.40**	**0.83**	**1.52**	**11.62**
	KNN		0.88	1.53	10.94	0.85	1.55	13.31
	GB		0.98	0.67	4.21	0.85	1.56	12.94

*The seven VIs were selected based on SHAP analysis (cumulative contribution > 90%). Bold values indicate the best predictive performance.

For PNA estimation, among the simple regression models, DTH achieved the best performance, with training R^2^ = 0.81 and testing R^2^ = 0.76, significantly outperforming other VIs (e.g., NDVI and NDRE, which had training R^2^ values of only 0.29–0.39). This indicates that the temporal progression of grain filling is a better indicator of PNA than single-temporal VIs. Multiple stepwise regression, which integrated all VIs, improved training R^2^ to 0.85, but testing R^2^ remained at 0.76, comparable to the DTH-based simple regression, suggesting some degree of overfitting. Machine learning models (MLP, KNN, and GB) substantially improved estimation accuracy. Compared to the best simple regression model (DTH), MLP reduced RMSE by 19.1% (from 1.88 to 1.52 g/m²) and rRMSE by 11.6% (from 13.15% to 11.62%) on the testing set. Among the machine learning models, testing R^2^ values reached 0.83–0.85, with RMSE decreasing to 1.52–1.56 g/m² and rRMSE ranging from 11.6% to 13.3%, indicating that nonlinear models can better capture the complex relationships between PNA and multiple predictors. The best estimation results for PNA were achieved by MLP (**Figure 7B**). PNA was more easily explained by DTH (temporal progression), whereas LNA showed stronger dependence on VIs. This may be related to the strong temporal pattern of PNA during grain filling, while LNA is more influenced by cultivar and N treatment effects. Machine learning models demonstrated substantial improvements over linear models, particularly for LNA estimation, where MLP increased testing R^2^ from 0.73 (multiple stepwise regression) to 0.89, and reduced rRMSE from 15.92% to 8.71%, representing a 45.3% reduction in relative error. Some overfitting was observed in multiple stepwise regression and certain machine learning models (e.g., GB for PNA achieved training R^2^ = 0.98 but testing R^2^ = 0.85), though testing accuracy remained within acceptable ranges.

### 4.3 Remote evaluation of rice NUtE by chlorophyll-related indices

**Figure 8:**
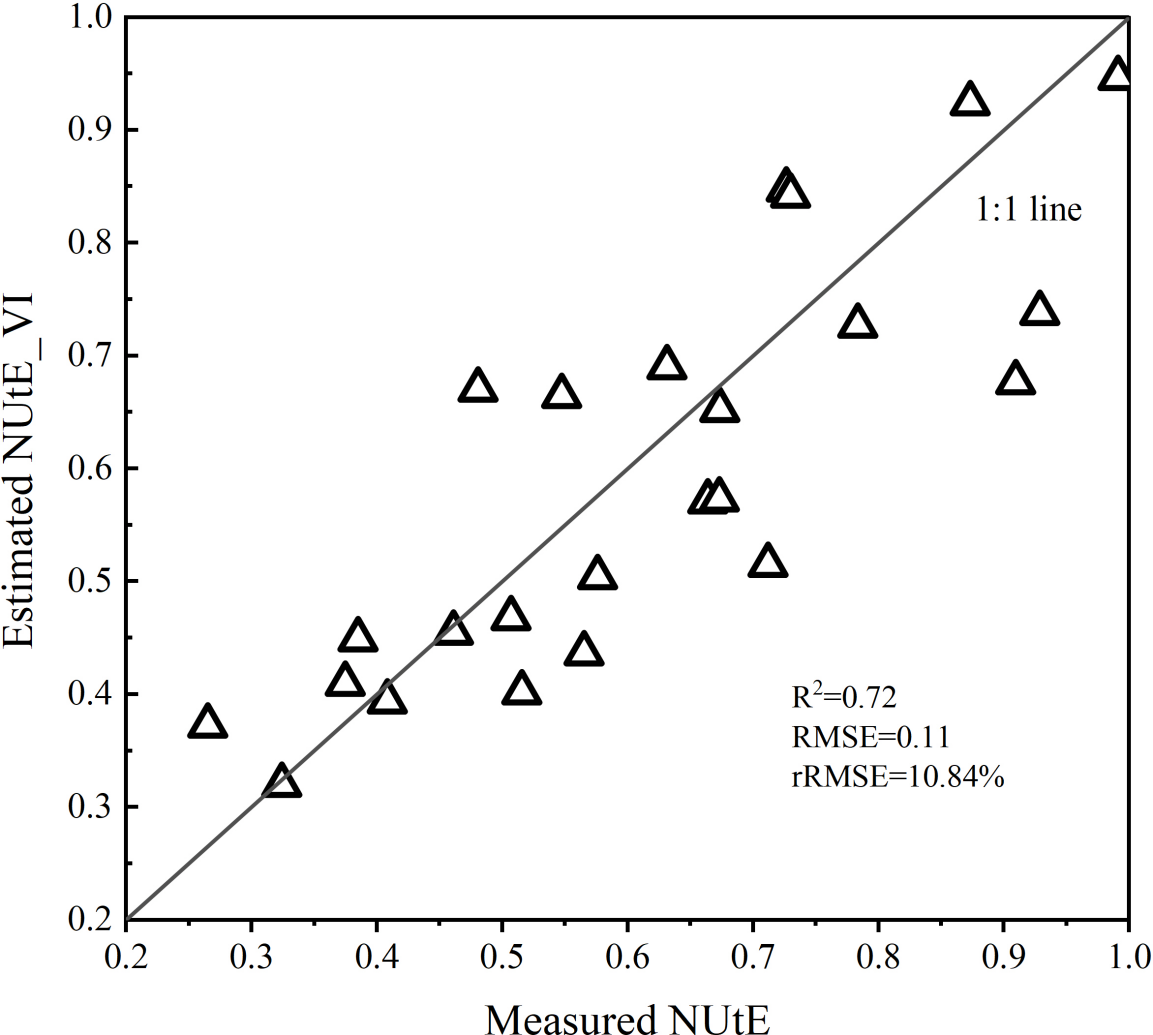
Relationship between estimated NUtE_VI and measured NUtE.

Since NUtE is closely related to rice leaf and panicle nitrogen content at key growth stages (see section 4.1) and rice leaf and panicle nitrogen content can be accurately estimated by chlorophyll-related indices (see section 4.2), this study developed a remote indicator for rice NUtE as the ratio of estimated ΔPNA_dough-heading_ to LNA_booting_ (Equation 19). The relationship between the estimated NUtE_VI and measured NUtE is shown in **Figure 8**. A significant positive correlation was observed, with R² = 0.72, RMSE = 0.11, and rRMSE = 10.84%. The points clustered closely around the 1:1 line, indicating that the NUtE_VI effectively captures the variation in NUtE. These results demonstrate that the NUtE_VI, constructed from remotely sensed LNA and ΔPNA, provides a reliable proxy for estimating NUtE in rice.

(19) $$NUtE\_VI=\frac{MLP_{△\text{PNAdough-heading}}(DTH,RVI,EVI2,RDVI,NDRE,NDVI,CI_{rededge})}{MLP_{LNA_{booting}}(MTCI,DTH,CI_{rededge},EVI2,NDRE,RDVI,RVI)}$$

Where $MLP_{△\text{PNAdough-heading}}(DTH,RVI,EVI2,RDVI,NDRE,NDVI,CI_{rededge})$ is the panicle nitrogen increment from heading to dough, estimated by MLP using remotely sensed indicators as a proxy of ΔPNA_dough-heading_ in Equation 18, and $MLP_{LNA_{booting}}(MTCI,DTH,CI_{rededge},EVI2,NDRE,RDVI,RVI)$is the leaf nitrogen accumulation at booting, estimated by MLP using remotely sensed indicators as a proxy of LNA_booting_ in Equation 18.

**Figure 9:**
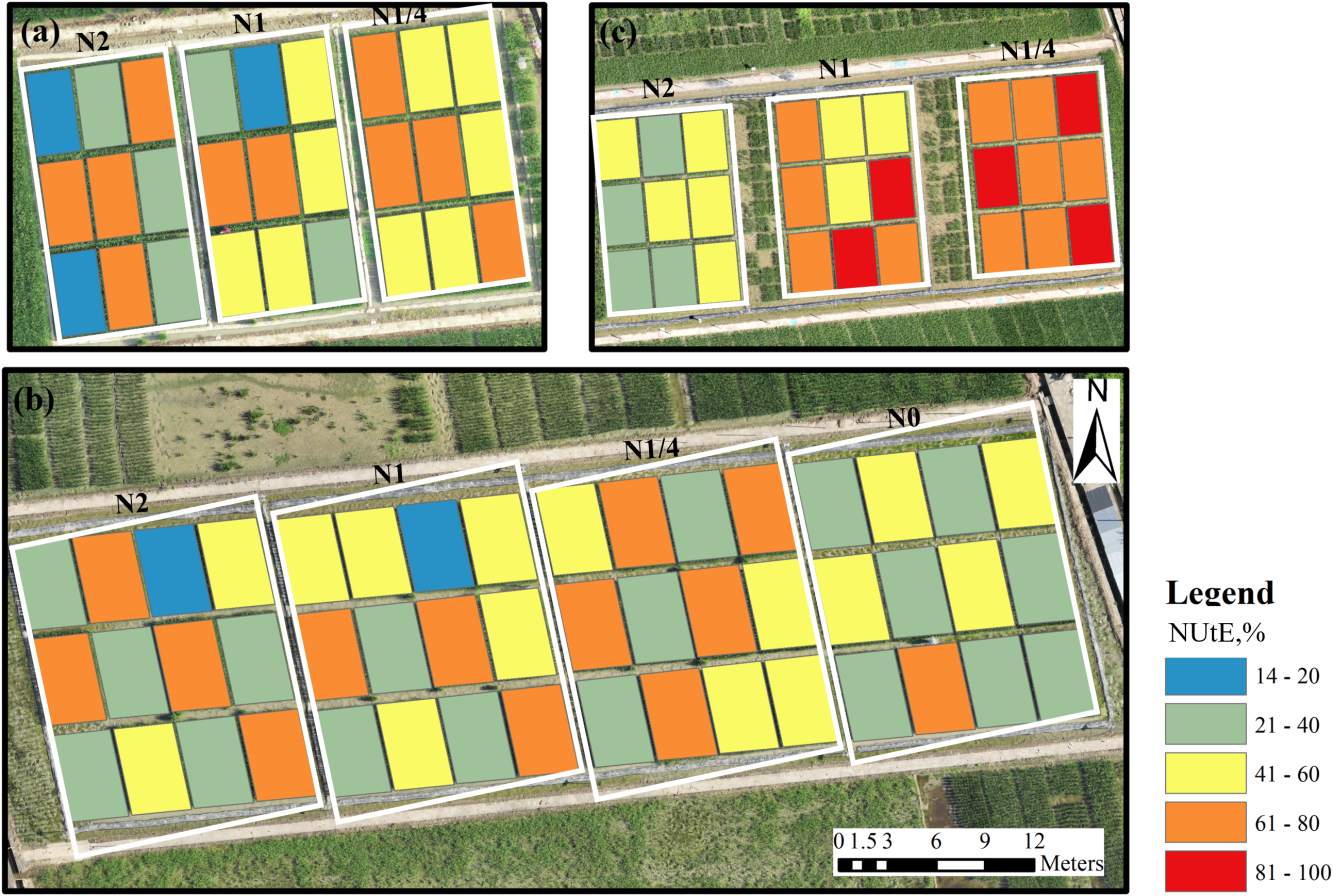
Spatial distribution of nitrogen utilization efficiency (NUtE) estimated by the NUtE-VI from 2022 to 2024. **(A)** 2022; **(B)** 2023; **(C)** 2024.

To evaluate the applicability of the NUtE_VI across different years and sites, the spatial distribution of estimated NUtE was mapped for the experimental fields from 2022 to 2024 (**Figure 9**). The spatial patterns revealed consistent variation across years, with higher NUtE values observed under low nitrogen treatment (N1/4, 36 kg/ha) and lower values under high nitrogen treatment (N2, 288 kg/ha). The temporal consistency of the spatial patterns suggests that the NUtE_VI captures stable differences in NUtE across the study area.

**Figure 10:**
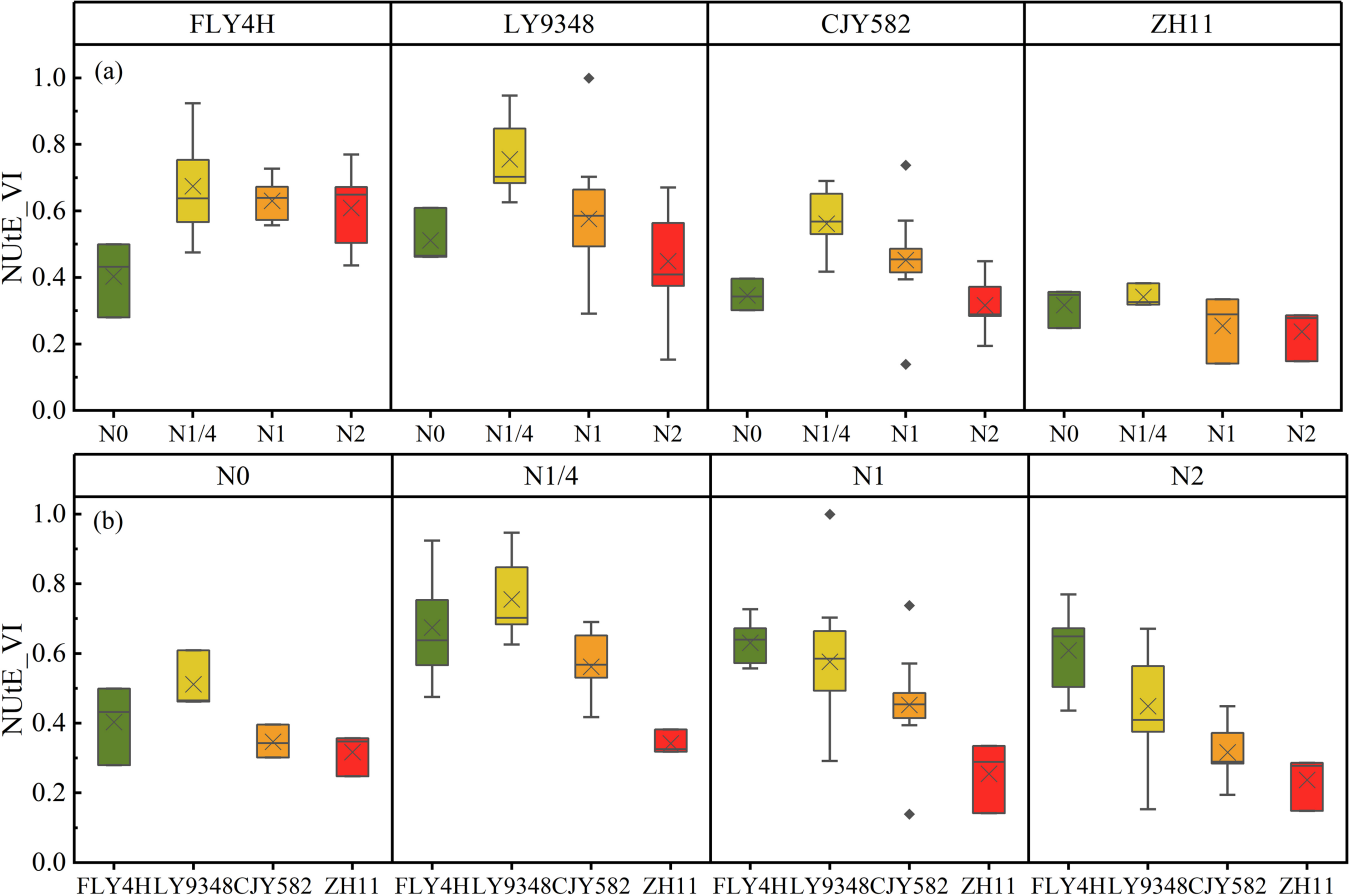
Comparison of NUtE_VI across cultivars and nitrogen treatments. **(A)** NUtE of different cultivars under varying nitrogen treatments; **(B)** NUtE across nitrogen treatments for each cultivar.

To further assess the sensitivity of NUtE_VI to genotypic variation and nitrogen management by comparing its values across four cultivars under four nitrogen treatments (**Figure 10**). The results revealed distinct patterns of cultivar performance and nitrogen responsiveness. Across nitrogen treatments (**Figure 10A**), indica rice (FLY4H and LY9348) consistently exhibited higher NUtE than japonica rice (CJY582 and ZH11). For FLY4H, NUtE was lowest under N0 (0 kg/ha) condition and increased substantially with nitrogen application, reaching a plateau from N1/4 (36 kg/ha) onward. In contrast, LY9348 showed a unimodal response, with peak NUtE at N1/4 followed by a progressive decline at higher nitrogen levels. A similar pattern was observed for the japonica rice (CJY582 and ZH11), both achieving maximum values at N1/4, with subsequent decreases under elevated nitrogen supply. Across cultivars (**Figure 10B**), LY9348 exhibited the highest NUtE under N0 and N1/4, followed by FLY4H, while ZH11 consistently ranked lowest. At N1 (144 kg/ha) and N2 (288 kg/ha), FLY4H surpassed LY9348 as the top performer, with LY9348 ranking second, followed by CJY582 and ZH11. This shift in cultivar ranking across nitrogen levels suggests differential nitrogen responsiveness among the tested cultivars.

## 5 Discussion

### 5.1 Biological basis of the development of NUtE-VI

The essence of NUtE lies in the efficiency of nitrogen remobilization from vegetative organs (leaves and stems) to reproductive organs (panicles) during the grain-filling period, and this key physiological process is inherently associated with dynamic changes in canopy spectral characteristics (Zheng et al., 2022). As panicles emerge and grain filling proceeds, nitrogen and photosynthetic assimilates stored in leaves and stems are gradually translocated to developing panicles, causing leaves to progressively turn yellow while panicles become increasingly plump and grains gradually turn yellow. During this process, leaf chlorophyll content declines, leading to an increase in canopy reflectance within the red edge region, a decrease in near-infrared reflectance, and weakened absorption in the visible spectrum (Fita et al., 2025; Impollonia et al., 2024; Peng et al., 2024). These distinct temporal dynamics of canopy reflectance are closely associated with the intensity and efficiency of nitrogen remobilization. Therefore, accurately capturing the temporal dynamics of canopy reflectance across critical growth stages provides a feasible and indirect means of assessing nitrogen remobilization efficiency in rice plants.

The construction of NUtE_VI is grounded in a fundamental physiological principle. the formation of nitrogen utilization efficiency (NUtE) in rice involves two temporally distinct processes: nitrogen accumulation during vegetative growth (source) and nitrogen remobilization during grain filling (sink). This temporal separation allows the two processes to be estimated independently using remote sensing. As shown in **Figure 3**, leaf nitrogen accumulation at booting stage (LNA_booting_) determines the total plant nitrogen pool (r = 0.95), while panicle nitrogen accumulation from heading to dough (ΔPNA_dough-heading_) determines final grain yield (r = 0.76). These processes occur at distinct growth stages and involve different organs, with corresponding canopy spectral responses that together provide the physiological basis for the NUtE_VI. Therefore, by remotely estimating LNA_booting_ and ΔPNA_dough-heading_ using VIs and their temporal dynamics, the ratio of these two components can be used as a proxy to indicate NUtE, enabling rapid and non-destructive assessment of rice nitrogen utilization efficiency.

During vegetative phase, both leaves and stems function as nitrogen storage pools, but leaves play a more prominent role. LNA increased progressively from tillering to booting, with its correlation with total plant nitrogen accumulation (TPNA) rising from 0.23 to 0.95. In contrast, stem nitrogen correlation peaked at late jointing and declined slightly thereafter. This pattern reflects the differential allocation of nitrogen in vegetative organs: late jointing is a critical period for stem elongation, during which substantial nitrogen is allocated to stems for structural development; after booting, nitrogen is preferentially stored in leaves in preparation for remobilization during grain filling (Ning et al., 2025). Thus, selecting LNA_booting_ as the indicator of the TPNA pool maximizes the representation of overall plant nitrogen status. After heading, the nitrogen dynamics of different organs exhibited a clear trade−off. Nitrogen accumulation in leaves and stems declined, while PNA increased, reflecting the remobilization of nitrogen from source to sink (Zhu et al., 2024). Notably, LNA increments shifted from positive to negative after heading to filling, whereas stem nitrogen increments remained consistently negative. This difference may be related to the distinct physiological roles of these organs: leaves serve both as nitrogen storage and photosynthetic sites, requiring sufficient nitrogen to sustain assimilate production during early grain filling; stems primarily function as structural support and storage organs, with their nitrogen being preferentially remobilized during grain filling (Li et al., 2025, 2026). The heading to dough interval represented the most active phase of nitrogen remobilization, consistent with the grain filling process (Fu et al., 2024). Therefore, selecting PNA during this interval as the indicator of nitrogen remobilization capacity is physiologically well−grounded.

The NUtE_VI, defined as the ratio of ΔPNA_dough-heading_ to LNA_booting_, has a clear physiological meaning, it represents the amount of panicle nitrogen accumulated per unit of total plant nitrogen, essentially capturing the nitrogen efficiency concept underlying NUtE. Compared with direct estimation of NUtE, this decomposition−reconstruction strategy offers two advantages. First, it breaks down a complex integrative indicator into two relatively independent physiological processes, each with distinct spectral response mechanisms, thereby reducing the complexity of remote sensing estimation. Second, the ratio form helps normalize differences in absolute nitrogen content across cultivars and nitrogen treatments, potentially enhancing the generalizability of the index across diverse conditions. Therefore, priority should be given to ensuring the estimation accuracy of the denominator (LNA_booting_). The ratio form tends to amplify prediction errors when the denominator is either intrinsically small in magnitude or subject to high uncertainty. Consequently, applications of NUtE_VI should avoid cases where LNA_booting_ falls below a reasonable range or carries large estimation errors, as these conditions can destabilize the index and compromise its interpretability. The strong correlation between NUtE_VI and measured NUtE (r = 0.81) validates the effectiveness of this construction strategy.

### 5.2 Chlorophyll-related spectral indices in response to organs nitrogen accumulation

This study revealed that canopy chlorophyll content (CCC) is positively correlated with LNA and negatively correlated with PNA (**Figure 5**). These results highlight the role of CCC as a bridge linking canopy spectra to nitrogen dynamics (Kang et al., 2025). As leaves constitute the major component of the canopy, their chlorophyll content directly determines canopy reflectance in the red−edge and near−infrared regions, establishing the spectral basis for the positive correlation between CCC and LNA. In contrast, The negative correlation between CCC and PNA reflects the physiological process of nitrogen remobilization during grain filling (Zhou et al., 2024). as leaf nitrogen is exported to panicles, leaf chlorophyll degrades, CCC declines, and panicle nitrogen accumulates. This trade−off relationship implies that monitoring temporal changes in CCC allows not only estimation of leaf nitrogen status but also indirect inference of panicle nitrogen accumulation dynamics, providing a physiological basis for simultaneously estimating LNA and PNA using multi−temporal remote sensing data.

SHAP analysis revealed distinct contribution patterns of VIs to LNA and PNA estimation (**Figure 6**). For LNA, all Chl-related VIs exhibited positive contributions, consistent with the well−established positive relationship between leaf nitrogen and chlorophyll content. Notably, MTCI showed the highest contribution, likely due to its high sensitivity to chlorophyll variation. The negative contribution of DTH reflected the effect of phenological progression: negative DTH values correspond to pre−heading (leaf nitrogen accumulation), while positive DTH values correspond to post−heading (leaf nitrogen decline). DCNI exhibited a negative contribution with the exception of a few samples, and its SHAP value ranked relatively low among all features. For PNA, DTH showed a distinctly positive contribution, indicating that grain filling progression is the primary driver of PNA, while most Chl-related VIs showed negative contributions, consistent with the negative CCC−PNA relationship. In contrast, DCNI showed a positive contribution to PNA estimation, likely reflecting that higher DCNI values indicate more advanced leaf senescence, indirectly indicating greater nitrogen remobilization to panicles. This contrast in contribution directions suggests that the same VI may play opposite roles in estimating different nitrogen-related traits, underscoring the importance of physiological interpretation in model application.

The opposite contribution directions of the same VI between LNA and PNA inversion can be explained by the fundamentally different physiological roles of leaf chlorophyll during vegetative and reproductive stages. During vegetative phase, chlorophyll serves as the primary light-harvesting pigment, and its content is closely coupled with leaf nitrogen because nitrogen is a major component of chlorophyll molecules and photosynthetic enzymes (e.g., Rubisco). Therefore, higher chlorophyll content (and thus higher VI values) directly indicates greater LNA, yielding positive contributions for LNA estimation. However, during grain filling, the source-sink relationship reverses. As nitrogen is remobilized from leaves to panicles, chlorophyll degrades and leaf nitrogen declines. Under this physiological context, a higher VI value at a given post-heading stage does not indicate higher leaf nitrogen; instead, it may reflect delayed senescence or slower nitrogen remobilization, which is associated with less PNA. Thus, the same VI that positively contributes to LNA during vegetative growth shows a negative contribution to PNA during grain filling. This contrast is not a statistical artifact but a direct spectral manifestation of the physiological trade-off between source maintenance and sink filling.

Machine learning models, particularly MLP, substantially outperformed linear models in estimating both LNA and PNA (**Table 4**). This advantage stems from two factors: first, spectral variables exhibit complex nonlinear relationships with nitrogen indicators that linear models cannot adequately capture; second, multiple VIs exhibit collinearity and interactions that machine learning models can automatically handle. Notably, the improvement in LNA estimation (rRMSE reduced from 15.92% to 8.71%) was greater than that for PNA (rRMSE reduced from 20.32% to 11.62%), likely because LNA has a more complex relationship with spectral variables, being more influenced by cultivar−by−nitrogen interactions. However, some machine learning models (e.g., GB) showed noticeable overfitting (training R² = 0.98, testing R² = 0.85), highlighting the need to balance model complexity and generalizability in practical applications.

PNA was more readily explained by DTH (temporal progression), whereas LNA showed stronger dependence on VIs. This difference likely arises from the distinct physiological formation mechanisms of the two indicators. PNA exhibits a strong temporal trajectory during grain filling, primarily driven by phenological progression, which can be well captured by a simple temporal variable such as DTH. In contrast, LNA is influenced by multiple factors. It is not only driven by phenological progression but also varies with cultivar differences in nitrogen uptake and storage capacity, as well as nitrogen supply levels. These complex interactions make LNA less predictable by a single temporal variable, requiring multispectral information to capture the combined effects of genotype, environment, and nitrogen management. This finding has practical implications for model selection. A simple DTH−based model may suffice for PNA, while full use of multispectral VIs is recommended for LNA. More importantly, this difference also explains why the decomposition−reconstruction strategy underlying NUtE-VI is effective. Breaking down complex NUtE into two relatively independent components, each estimated with simpler models, yields better accuracy than directly estimating NUtE.

The NUtE_VI, constructed from optimal models for LNA_booting_ and ΔPNA_dough-heading_, showed a strong positive correlation with measured NUtE (R² = 0.72, rRMSE = 10.84%) (**Figure 8**). This result validates the effectiveness of the decomposition−reconstruction strategy, in which two physiologically meaningful intermediate variables are estimated separately and then combined to form the final index. Moreover, the construction method of NUtE_VI has a clear physiological basis, which may enhance model transferability across years, cultivars, and nitrogen treatments. The physiological mechanisms underlying the intermediate variables LNA_booting_ and ΔPNA_dough-heading_ are stable across conditions, whereas purely statistical models often fail to generalize.

### 5.3 Performance of using NUtE-VI for evaluation NUtE for different rice cultivars

The spatial distribution of NUtE estimated by NUtE_VI showed consistent patterns across years (**Figure 9**), with higher values under low nitrogen (N1/4, 36 kg/ha) and lower values under high nitrogen (N2, 288 kg/ha), consistent with the physiological response of rice to nitrogen availability. The temporal consistency across years indicates that NUtE-VI captures stable differences in NUtE rather than year−specific fluctuations, a crucial advantage over traditional empirical models that often fail under varying climatic conditions.

The NUtE_VI effectively distinguished NUtE differences between indica and japonica rice (**Figure 10A**). Across all nitrogen treatments, indica rice (FLY4H and LY9348) consistently exhibited higher NUtE than japonica rice (CJY582 and ZH11), consistent with the physiological characteristics of indica rice, which typically has higher nitrogen remobilization efficiency (Gao et al., 2019; Xiong et al., 2025; Zhang et al., 2025). Notably, FLY4H and LY9348 exhibited contrasting nitrogen response patterns. LY9348 showed a unimodal response, peaking at N1/4 (36 kg/ha) and declining at higher nitrogen levels, characteristic of a low−nitrogen−efficient cultivar. This is consistent with previous studies demonstrating that LY9348 exhibits higher NUtE under low nitrogen conditions, with its glutamine synthetase regulatory networks specifically activated under nitrogen deficiency to enhance nitrogen uptake and remobilization (Liang et al., 2021a, 2021b). In contrast, FLY4H showed a plateau response, with NUtE increasing from N0 to N1/4 and stabilizing thereafter, characteristic of a high−nitrogen−efficient cultivar, also supported by previous findings (Nie et al., 2022; Xiao et al., 2024). This difference reflects distinct regulatory mechanisms of nitrogen translocation in these two cultivars, LY9348 relies on specific gene networks activated under low nitrogen conditions, whereas FLY4H maintains higher remobilization efficiency under high nitrogen supply. The japonica rice CJY582 and ZH11 exhibited similar unimodal responses to LY9348, though with lower overall NUtE values, reflecting fundamental differences in nitrogen utilization strategies between indica and japonica rice.

The shift in cultivar ranking across nitrogen treatments (**Figure 10B**) further validated the distinct nitrogen efficiency types of LY9348 and FLY4H. Under low nitrogen conditions (N0 and N1/4), LY9348 outperformed all other cultivars, confirming its low−nitrogen−efficient nature, whereas under high nitrogen conditions (N1 and N2), FLY4H emerged as the top performer, confirming its high−nitrogen−efficient nature (Liang et al., 2021b). This ranking shift suggests that selection of nitrogen−efficient cultivars should be tailored to specific nitrogen management scenarios, cultivars that perform well under low nitrogen input may not necessarily be superior under high nitrogen conditions. This finding has important implications for breeding strategies, different breeding targets require different cultivar selections depending on the intended nitrogen management regime. For example, LY9348 is a better choice for low−input sustainable production systems, whereas FLY4H is more suitable for conventional nitrogen management.

The consistent performance of the NUtE_VI across cultivars, nitrogen treatments, and years validates the effectiveness of its physiologically based construction strategy. The index accurately captured the fundamental differences in nitrogen utilization strategies between indica and japonica rice, as well as the distinct nitrogen efficiency types of LY9348 (low−N efficient) and FLY4H (high−N efficient). Unlike empirical models that directly estimate NUtE, the NUtE_VI enhances generalizability by decomposing NUtE into two physiologically meaningful intermediate variables (source and sink), whose underlying mechanisms remain relatively stable across conditions, while direct statistical relationships often vary with environmental conditions. The spatial consistency across years, accurate capture of cultivar differences, and reasonable responses to nitrogen treatments collectively demonstrate the reliability and generalizability of the NUtE_VI for estimating NUtE under diverse conditions. This provides an effective remote sensing tool for high throughput screening of nitrogen−efficient cultivars and precision nitrogen management. Notably, japonica rice consistently exhibited lower NUtE than indica rice under the same nitrogen conditions. For practical cultivation, LY9348 is suitable for low−fertility soils due to its ability to maintain relatively high yield under low nitrogen supply, whereas FLY4H is better suited for fertile soils where its high yield potential can be fully realized. This study further demonstrates that remote sensing enables large−scale, cost−effective, and non−destructive evaluation of cultivar performance across different nitrogen regimes, highlighting the substantial potential of remote sensing−assisted precision agriculture.

### 5.4 Limitations and perspectives

Despite the promising performance of NUtE_VI in assessing rice NUtE, several limitations should be acknowledged regarding its applicability. (1) the development and validation of NUtE_VI were based on limited four rice cultivars, including both indica and japonica types, which represent the major rice ecotypes cultivated in the middle and lower reaches of the Yangtze River. While these cultivars capture a substantial range of NUtE diversity, the generalizability of this index to other cultivar groups, such as hybrid rice with distinct source-sink characteristics or upland rice cultivars, remains to be tested. (2) the current study was conducted under controlled nitrogen gradient conditions with adequate water supply and no major pest or disease pressure. The performance of NUtE_VI under suboptimal conditions, such as drought, waterlogging, heat stress, or cold stress during grain filling, is unknown. Extreme weather events may alter canopy structure and spectral responses in ways that deviate from the physiological assumptions underlying NUtE_VI. (3) the decomposition-reconstruction strategy relies on the accurate estimation of LNA_booting_ and ΔPNA_dough-heading_ using multi-temporal UAV imagery. In cropping systems with different planting densities, row configurations, or multiple harvests (e.g., ratoon rice), the optimal timing for capturing these two components may shift, requiring site-specific calibration.

The present dataset also suggests two points that may be valuable for future research. First, the strong correlation between LNA_booting_ and TPNA (r = 0.95) suggests that booting stage LNA could serve as a reliable proxy for total plant nitrogen pool across diverse cultivars and nitrogen treatments, potentially simplifying field sampling protocols. Second, the temporal window from heading to dough stage consistently captured the most active phase of nitrogen remobilization, implying that spectral monitoring during this 10–35 days interval may be sufficient for assessing NUtE in indica-japonica cultivation systems.

Future work should extend the validation of NUtE_VI to a broader range of genetic materials, cropping systems, and environmental conditions. In particular, the integration of thermal and radar remote sensing data should be explored to improve estimation robustness under adverse weather conditions. Additionally, developing automated pipelines for timely extraction of LNA_booting_ and ΔPNA_dough-heading_ from UAV image time series would facilitate the practical application of NUtE_VI in large-scale breeding programs and precision nitrogen management.

## 6 Conclusion

This study developed a mechanically based remote sensing index, NUtE_VI, for estimating nitrogen utilization efficiency (NUtE) in rice. The index integrates two key physiological indicators: leaf nitrogen accumulation at booting stage, representing the total plant nitrogen pool, and panicle nitrogen accumulation from heading to dough, representing nitrogen remobilization capacity. Using multispectral imagery and machine learning, both indicators were estimated with high accuracy, and NUtE_VI showed a strong correlation with measured NUtE (R² = 0.72, rRMSE = 10.84%). The index captured expected patterns across nitrogen treatments and cultivars, with indica rice showing higher NUtE than japonica rice, and successfully distinguished LY9348 (low nitrogen efficiency) from FLY4H (high nitrogen efficiency). These results demonstrate that NUtE-VI provides a reliable, physiologically based proxy for NUtE with promising generalizability across years, cultivars, and nitrogen treatments. Future research should focus on developing automated phenological stage extraction methods and evaluating the index’s performance under more diverse ecological conditions and stress environments.

## Data Availability

The data analyzed in this study is subject to the following licenses/restrictions: As the original data contain proprietary or undisclosed cultivar information, the data are available upon the approval of the corresponding author. Requests to access these datasets should be directed to ypeng@whu.edu.cn.
